# NPTX2 promotes colorectal cancer growth and liver metastasis by the activation of the canonical Wnt/β-catenin pathway via FZD6

**DOI:** 10.1038/s41419-019-1467-7

**Published:** 2019-03-04

**Authors:** Chunjie Xu, Guangang Tian, Chunhui Jiang, Hanbing Xue, Manzila Kuerbanjiang, Longci Sun, Lei Gu, Hong Zhou, Ye Liu, Zhigang Zhang, Qing Xu

**Affiliations:** 1 Department of Gastrointestinal Surgery, Renji Hospital, School of Medicine, Shanghai Jiao Tong University, 160 Pujian Road, Shanghai, 200127 P.R. China; 2 State Key Laboratory of Oncogenes and Related Genes, Shanghai Cancer Institute, Renji Hospital, School of Medicine, Shanghai Jiaotong University, Shanghai, P.R. China; 3 Division of Gastroenterology and Hepatology; Key Laboratory of Gastroenterology and Hepatology, Ministry of Health; Renji Hospital, School of Medicine, Shanghai Jiao Tong University; Shanghai Institute of Digestive Disease, 145 Middle Shandong Road, Shanghai, 200001 P.R. China

## Abstract

Accumulating evidence from clinical and epidemiological studies has highlighted the close correlation between the individual risk of cancer and nervous system diseases. The expression of neuronal pentraxin 2 (NPTX2) is absent in Alzheimer’s disease, anxiety, and depression. Herein, we found that NPTX2 mRNA and protein expression was significantly upregulated in colorectal carcinoma (CRC). NPTX2 expression level gradually increased with CRC progression and was closely associated with poor prognosis. In vitro and in vivo studies demonstrated that NPTX2 promoted CRC proliferation and metastasis through the activation of the Wnt/β-catenin signaling pathway. As NPTX2 receptors are absent on CRC cells, NPTX2 was shown to physically interact with frizzled class receptor 6 (FZD6) to promote β-catenin translocation into the cell nucleus, resulting in an increase in the expression of MYC, cyclin D1, snail, and N-cadherin along with a decrease in the expression of E-cadherin. Knockdown of FZD6 expression with a small-interfering RNA almost completely reversed the proliferative effects of NPTX2 on CRC development. In conclusion, NPTX2, a molecule related to nervous system diseases, promotes CRC cell proliferation and metastasis through the activation of the Wnt/β-catenin pathway via direct interaction with FZD6.

## Introduction

Colorectal carcinoma (CRC) is one of the most common malignant tumors of the gastrointestinal tract and ranks fourth in terms of incidence and mortality associated with human cancers^1,2^. In general, early-stage CRC, including stage 1 and stage 2, has a relatively good prognosis and 5-year survival rate, which could reach 70%. The prognosis of patients becomes extremely poor with the progress of CRC, especially in stage 4^3^. The main reason for treatment failure is metastasis, which is responsible for 90% of cancer-related mortality^4^. The incidence of liver metastasis was most common in CRC metastasis and reached up to 50%. However, very limited clinical prevention and treatment options are available for liver metastases. Therefore, there is an urgent need to explore the mechanism underlying CRC liver metastasis to improve our understanding of the pathology as well as to design potential therapeutic strategies.

Several clinical and epidemiological studies have revealed the close correlation between individual risk of cancer and nervous system diseases^5,6^. While some studies have described a positive correlation, others have suggested an inverse correlation^7^. Therefore, further studies are warranted to address this discrepancy.

Neuronal pentraxin 2 (NPTX2) is a member of the neuronal pentraxin family and is essential for synapse formation^8^. Pentraxins could be divided into short pentraxin and long pentraxin based on their lengths. NPTX2 belongs to the long pentraxin group and plays a role in host immunity and acute inflammation. It is closely associated with central nervous system diseases, such as Parkinson’s and Alzheimer’s diseases^9^. Recent studies have found that the abnormal upregulation in NPTX2 expression was correlated with the proliferation and metastasis of clear cell renal cell carcinoma and neuroblastoma^10,11^. However, whether NPTX2 is involved in CRC progression and metastasis, the possible underlying mechanism remains unclear.

In the present study, we found NPTX2 expression was upregulated in CRC, which promotes colorectal cancer growth and liver metastasis by the activation of the canonical Wnt/β-catenin pathway via FZD6.

## Materials and methods

### Patients and samples

A total of 392 paraffin sections of CRC tissues and adjacent paired non-cancerous tissues, including 30 liver metastasis tissues, were collected to design a tissue array chip from the Department of Gastrointestinal Surgery, Renji Hospital, School of Medicine, Shanghai Jiao Tong University. All patients with CRC underwent surgery at the Department of Gastrointestinal Surgery, Renji Hospital, School of Medicine, Shanghai Jiao Tong University between January 2014 and January 2016. The study was approved by the Research Ethics Committee of Renji Hospital and carried out in accordance with ethical standards as formulated in the Helsinki Declaration. Informed consents were provided by all patients.

### Cell culture

SW480 and LoVo (human CRC cell lines) cells were obtained from the Cell Bank of the Chinese Academy of Sciences (Shanghai, China). All cell lines were cultured in the Dulbecco’s modified Eagle’s medium (DMEM) supplemented with 10% fetal bovine serum (FBS) and 1% penicillin and streptomycin.

### Small-interfering RNA (siRNA) transfection

The siRNAs for NPTX2 and frizzled class receptor 6 (FZD6) were purchased from GenePharma (Shanghai GenePharma Co., Ltd., Shanghai, China). Sequences are shown in Table S1, and experimental method was performed as previously described^12^.

### Lentivirus transfection

Full-length human NPTX2 cDNA was transfected into CRC cell lines using a lentivirus to generate Lentivirus-NPTX2 (NPTX2-HA). Lentivirus-NC was used as the negative control (NPTX2-vector). In addition, one short-hairpin RNA (shRNA) sequence against NPTX2 was transfected into CRC cell lines to generate shRNA-NPTX2, while shNC-NPTX2 was used as the negative control. The sequences of shRNA-NPTX2 and shNC-NPTX2 were GCTCATCAACGACAAGGTTGC and TTCTCCGAACGTGTCACGT, respectively.

### RNA isolation and real-time quantitative polymerase chain reaction (RT-qPCR)

Trizol was used to extract RNA, and the total RNA was reverse transcribed to cDNA by PrimeScript^TM^ (Takara Biomedical Technology (Beijing) Co., Ltd.). We used 18S RNA as an internal control. The sequences of the primers used are shown in Table S1. The relative expression of the target gene was calculated by the −△△Ct method.

### Western blot analysis

Total protein was extracted using radioimmunoprecipitation assay (RIPA) buffer supplemented with 1% protease inhibitors and phosphatase inhibitors. Protein concentration was measured with the bicinchoninic acid (BCA) assay. Western blot analysis was performed as previously described^12^. NPTX2 (ab69858, Abcam), FZD6 (ab150545, ab98933, Abcam), β-catenin (ab32572, Abcam), MYC (ab39688, Abcam), cyclin D1 (CCND1; ab16663, Abcam), snail-1 (ab53519, Abcam), N-cadherin (ab76011, Abcam), E-cadherin (ab1416, Abcam), Ki-67, and proliferating cell nuclear antigen (PCNA; Proteintech Group, Inc.) primary antibodies were used. Horseradish peroxidase (HRP)-conjugated Affinipure goat anti-rabbit IgG (H + L) and HRP-conjugated Affinipure goat anti-mouse IgG (H + L) were obtained from Proteintech Group, Inc (Jackson).

### Immunohistochemistry (IHC)

All tissues were paraffin-embedded and cut into 4-μm-thick sections. All sections were dewaxed with xylene and hydrated with alcohol. Sodium citrate was used for antigen retrieval, and 0.3% hydrogen peroxide (H_2_O_2_) was used to block endogenous peroxidase. After blocking non-specific sites with bovine serum albumin (BSA), all the sections were incubated with an appropriate primary antibody and secondary antibody. We used 3,3′-diaminobenzidine (DAB) kit for visualization, and hematoxylin was used to stain nuclei. All the sections were dehydrated with alcohol and sealed with neutral resin. IHC staining score was calculated based on pixel intensity; staining was scored as follows as per the staining intensity: no staining, 1; weak staining, 2; moderate staining, 3; and strong staining, 4^13^.

### Cellular immunofluorescence

A 12-well chamber was used for cellular immunofluorescence assay (IBiDi). Each well was seeded with 1.5 × 10^4^ cells. After 1–2 days, the cells were fixed with 4% paraformaldehyde and treated with 0.2% Triton X-100 for 1 min. We used 1% bovine serum albumin (BSA) to block nonspecific binding sites. After incubation with primary and secondary antibodies, the tissue sections were stained with 4′,6-diamidino-2-phenylindole (DAPI) for 30 min and covered with a coverslip. Cellular immunofluorescence was observed through a fluorescence upright microscope and confocal microscope.

### Animal model

To generate a xenograft model, 5 × 10^6^ SW480 cells were injected into the left armpit of each null mouse. After 4 weeks, all null mice were killed and the xenograft tumors were excised. The tumor volume and weight were measured, and all tissues were fixed with 4% paraformaldehyde. To generate a liver metastasis model, all nude mice were anesthetized with 0.5% pentobarbital. The abdominal cavity was opened and 1 × 10^6^ SW480 cells/null mouse were injected into the spleen. After 2 weeks, the mice were killed and the liver metastasis tissues were excised. All tissues were fixed with 4% paraformaldehyde.

For the generation of an orthotopic model of CRC, all nude mice were anesthetized with 0.5% pentobarbital. After opening the abdominal cavity, 1 × 10^6^ SW480 cells/null mouse were injected into the ileocecum. After 4 weeks, the mice were killed and the tumor tissues were excised and weighed. All tissues were fixed with 4% paraformaldehyde. All animal experiments were approved by the Research Ethics Committee of Renji Hospital and adhered to the local or national requirements for the care and use of laboratory animals.

### Luciferase reporter assay

Sh-NPTX2 and control CRC cells were seeded onto 96-well plates and transfected with a mixture of 100 ng of TOP (TCF reporter plasmid) reporter plasmid (Wnt/β-catenin signaling) and 10 ng of *Renilla* as per the protocol recommended in Lipofectamine 2000 transfection system. After 24 h, firefly and *Renilla* luciferase activities were measured using the dual-luciferase reporter assay system (Promega, Madison, WI), as per the recommended protocol.

### Coimmunoprecipitation (Co-IP)

Total protein was extracted from CRC cells and incubated with appropriate primary antibody overnight, followed by the addition of protein A-Sepharose beads. After extensive washing, the precipitates were subjected to western blotting for the detection of the interacting proteins. Normal rabbit IgG served as a negative control. Anti-hemagglutinin was purchased from Medical & Biological Laboratories (Nagoya, Japan). Anti-NPTX2 was obtained from Proteintech Group, Inc, while anti-FZD6 was supplied by Cell Signaling Technology, Inc.

### Statistical analysis

Measurement data were presented as the mean ± standard deviation (SD). Statistical analyses were conducted by SPSS 20.0 (Chicago, IL, USA) and GraphPad Prism 5 software. Correlation of NPTX2 expression with categorical clinical variables in patients with CRC was evaluated with chi square analysis or Student’s *t* test. Measurement data, such as age and tumor size, were evaluated with Student’s *t* test, while categorical variables and ranked data, such as sex, T stage, lymph node invasion, and distant metastasis, were analyzed with chi square test. Spearman’s rank correlation was used for the analysis of two-way ordered categorical data. Survival curves were generated using the Kaplan–Meier method and analyzed by the log-rank test. Statistical significance was accepted at *P* < 0.05.

## Results

### NPTX2 is overexpressed in tumor tissues and its expression correlates with poor CRC prognosis

**Fig. 1:**
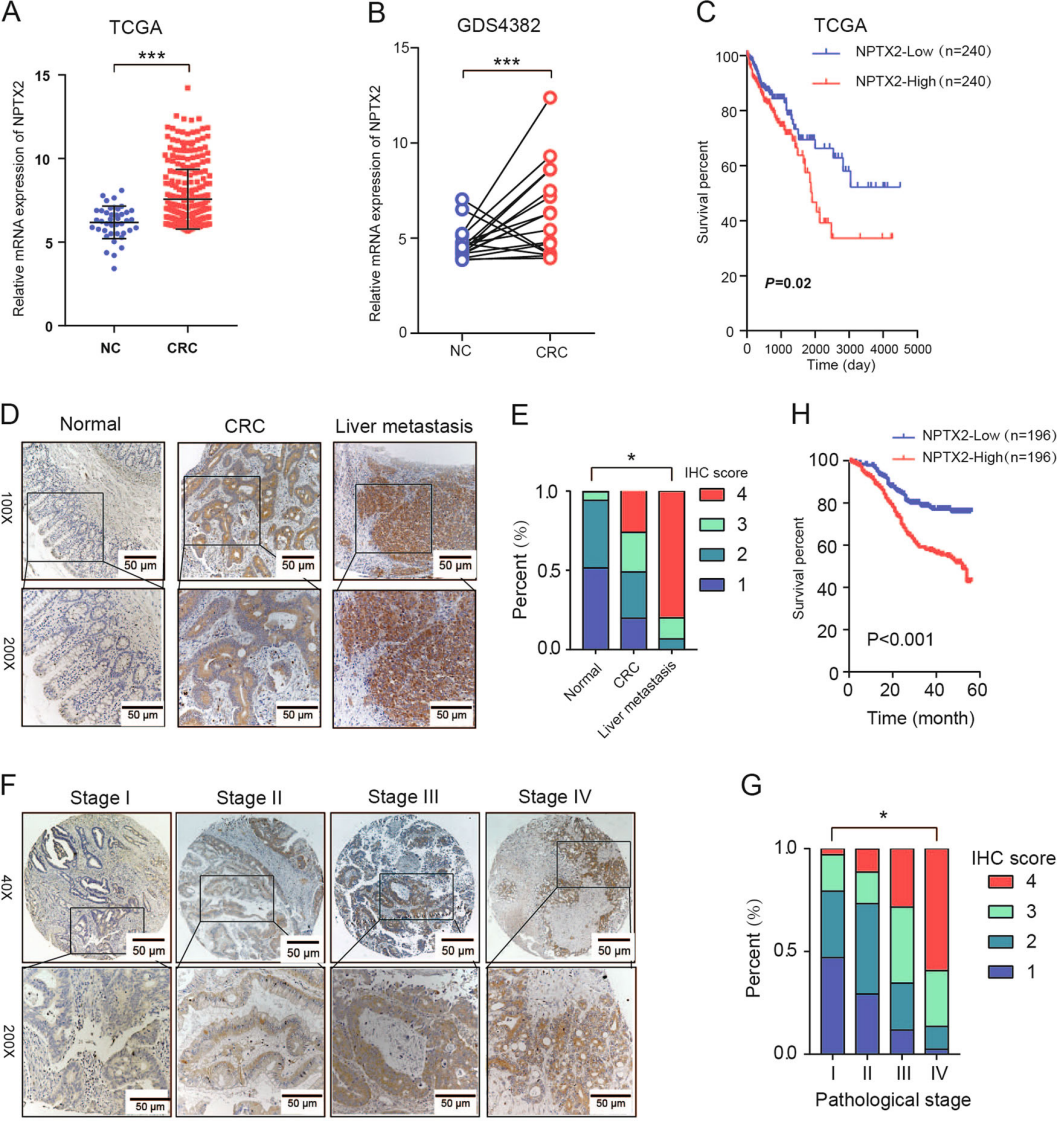
NPTX2 expression is upregulated in CRC tissues and correlated with patient prognosis. **a** The mRNA expression of NPTX2 in CRC and adjacent noncancerous tissues, as analyzed from TCGA dataset (Student’s *t* test). **b** The mRNA expression of NPTX2 in CRC and adjacent noncancerous tissues, as analyzed from GEO dataset (Student’s *t* test). **c** Overall survival analysis based on the mRNA expression of NPTX2 from TCGA dataset (Kaplan–Meier method and log-rank test). **d**, **e** The protein expression of NPTX2 in normal, CRC, and liver metastasis tissues in tissue microarray (392 cases of normal tissues, 392 matched cases of CRC tissues, and 30 cases of liver metastasis) (Spearman’s rank correlation). **f**, **g** NPTX2 protein expression at different pathological stages in CRC tissues from tissue microarray (34 cases of stage I, 150 cases of stage II, 127 cases of stage III, and 81 cases of stage IV CRC) (Spearman’s rank correlation). **h** Overall survival analysis of the protein expression of NPTX2 based on the prognostic information of patients with CRC from tissue microarray data (Kaplan–Meier method and log-rank test). Measurement data were presented as the mean ± SD. *P < 0.05; *** *P* < 0.0001

The gene expression data from The Cancer Genome Atlas (TCGA) and Gene Expression Omnibus (GEO) datasets showed that *NPTX2* mRNA expression was significantly increased in CRC tissues as compared with normal tissues (Fig. 1a, b). The patients with high *NPTX2* mRNA expression showed an obviously shorter overall survival than those with low *NPTX2* expression (Fig. 1c).

To evaluate the protein level of NPTX2 in CRC tissues, a tissue microarray with 392 cases of cancer and corresponding adjacent non-cancerous tissues was generated to detect the expression level of NPTX2 protein. The results of the microarray analysis showed that the expression of NPTX2 protein was significantly increased in CRC tissues as compared with adjacent noncancerous tissues. NPTX2 protein expression was obviously higher in liver metastasis than in the corresponding primary CRC (Fig. 1d, e). Further analysis showed that NPTX2 protein expression was gradually upregulated with an increase in T stages (Fig. S1A) and pathological stages (Fig. 1f, g). In addition, NPTX2 expression was significantly upregulated in CRC with lymph node invasion as compared with those without lymph node invasion; no difference was observed in NPTX2 protein expression between different lymph node invasion stages (Fig. S1B).

To investigate the clinical significance of NPTX2 in CRC, we analyzed NPTX2 expression with respect to various pathological parameters in 392 patients with CRC. The results showed that NPTX2 expression in CRC tissues was closely related to T stage, lymph node invasion, distant metastasis, and pathological stage (Table 1 and Table S2). Furthermore, survival analysis based on the expression level of NPTX2 revealed the significant decrease in the survival rate of patients with CRC that showed high NPTX2 expression as compared with those with low NPTX2 expression (Fig. 1h).

**Table 1:** The relationship between NPTX2 protein expression and clinical features in CRC from a tissue microarray

	NPTX2		*P-*value
	Low (*n * = 196)	High (*n* = 196)	
Age (year)	59.48 ± 8.56	60.64 ± 7.45	0.153
Sex			
Male	105	127	0.258
Female	81	79	
Tumor size (cm)	4.67 ± 1.54	7.39 ± 2.16	** <0.001**
T stage			
T1	9	2	
T2	29	7	** <0.001**
T3	43	51	
T4	115	136	
Lymph node invasion			
Yes	57	124	**<** **0.001**
No	139	72	
Distant metastasis			
Yes	22	59	**<** **0.001**
No	174	137	

In Table 1, as measurement data, like age and tumor size, Students’ *t* test was used for statistical analysis; as categorical variables or ranked data, Chi - square analysis was applied to statistical analysis

The significance bold value of tumor size is 8.14 x 10^−38^

The significance bold value of T stage is 0.000145

The significance bold value of lymph node invasion is 1.14 x 10^−11^

The significance bold value of distance metastasis is 3.92 x 10^−6^

### Knockdown of NPTX2 expression suppresses cell proliferation and migration in vitro and in vivo

**Fig. 2:**
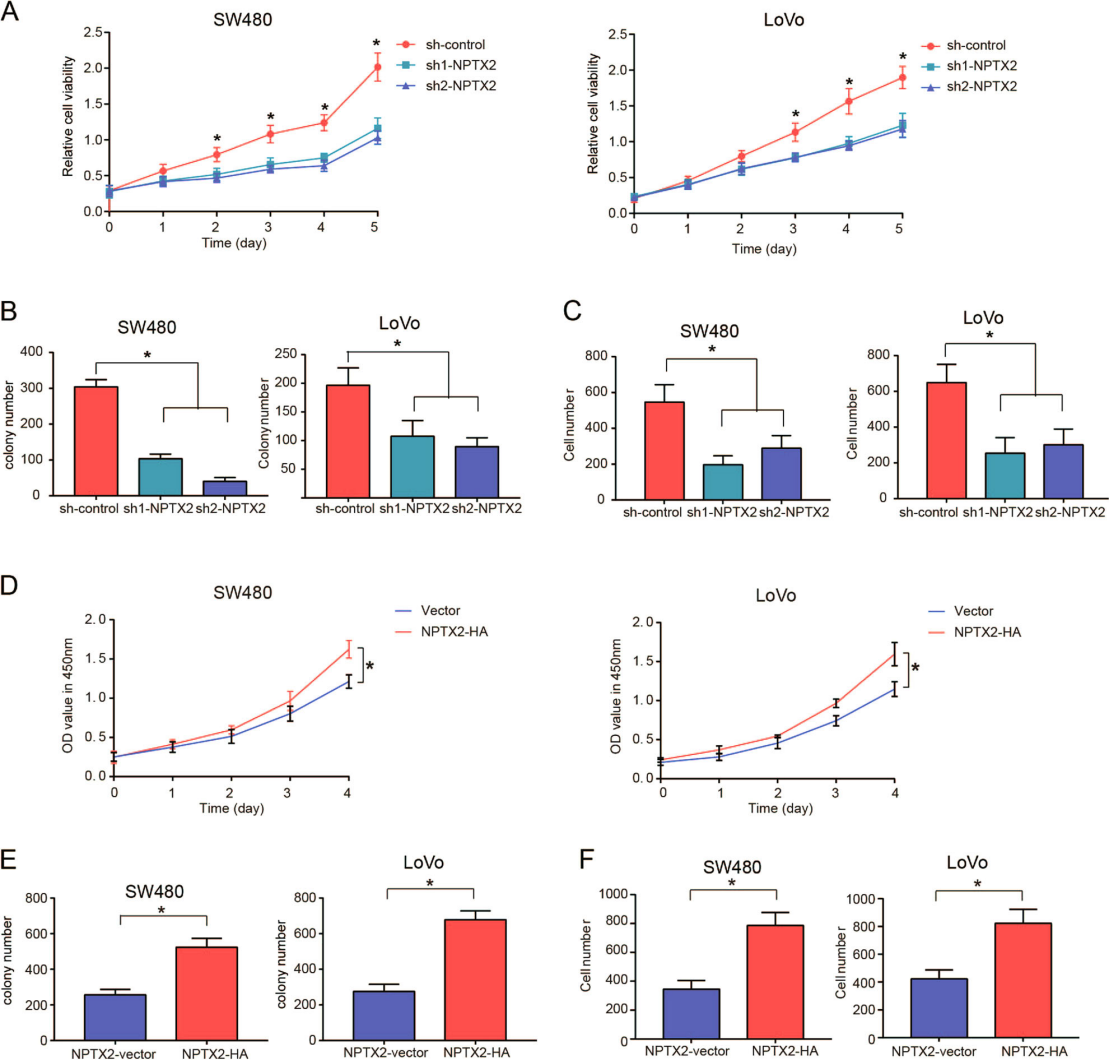
Knockdown of NPTX2 expression suppresses the viability, colony formation, and migration of CRC cells. **a** The viability of SW480 and LoVo cells transfected with sh-NPTX2 or sh-control, as analyzed with CCK-8 assay (Student’s *t* test). **b** Colony-formation ability of SW480 and LoVo cells transfected with sh-NPTX2 or sh-control (Student’s *t* test). **c** Cell-migration ability of SW480 and LoVo cells transfected with sh-NPTX2 or sh-control (Student’s *t* test). **d** The viability of SW480 and LoVo cells transfected with vector or NPTX2-HA, as detected with CCK-8 assay (Student’s *t* test). **e** Colony-formation ability of SW480 and LoVo cells transfected with vector or NPTX2-HA (Student’s *t* test). **f** Cell-migration ability of SW480 and LoVo cells transfected with vector or NPTX2-HA (Student’s *t* test). All experiments were performed in triplicates. Measurement data were presented as the mean ± SD. *P < 0.05

To clarity the functional role of NPTX2 in CRC, we knocked down and overexpressed NPTX2 in two CRC cell lines, SW480 and LoVo (Fig. S2A–S2C). The results showed that the knockdown of NPTX2 expression significantly inhibited cell proliferation and cell migration abilities of both SW480 and LoVo cells (Fig. 2a–c and Fig. S2D, S2E). NPTX2 overexpression significantly enhanced cell proliferation and cell migration abilities of both SW480 and LoVo cells (Fig. 2d–f and Fig. S2F, S2G).

**Fig. 3:**
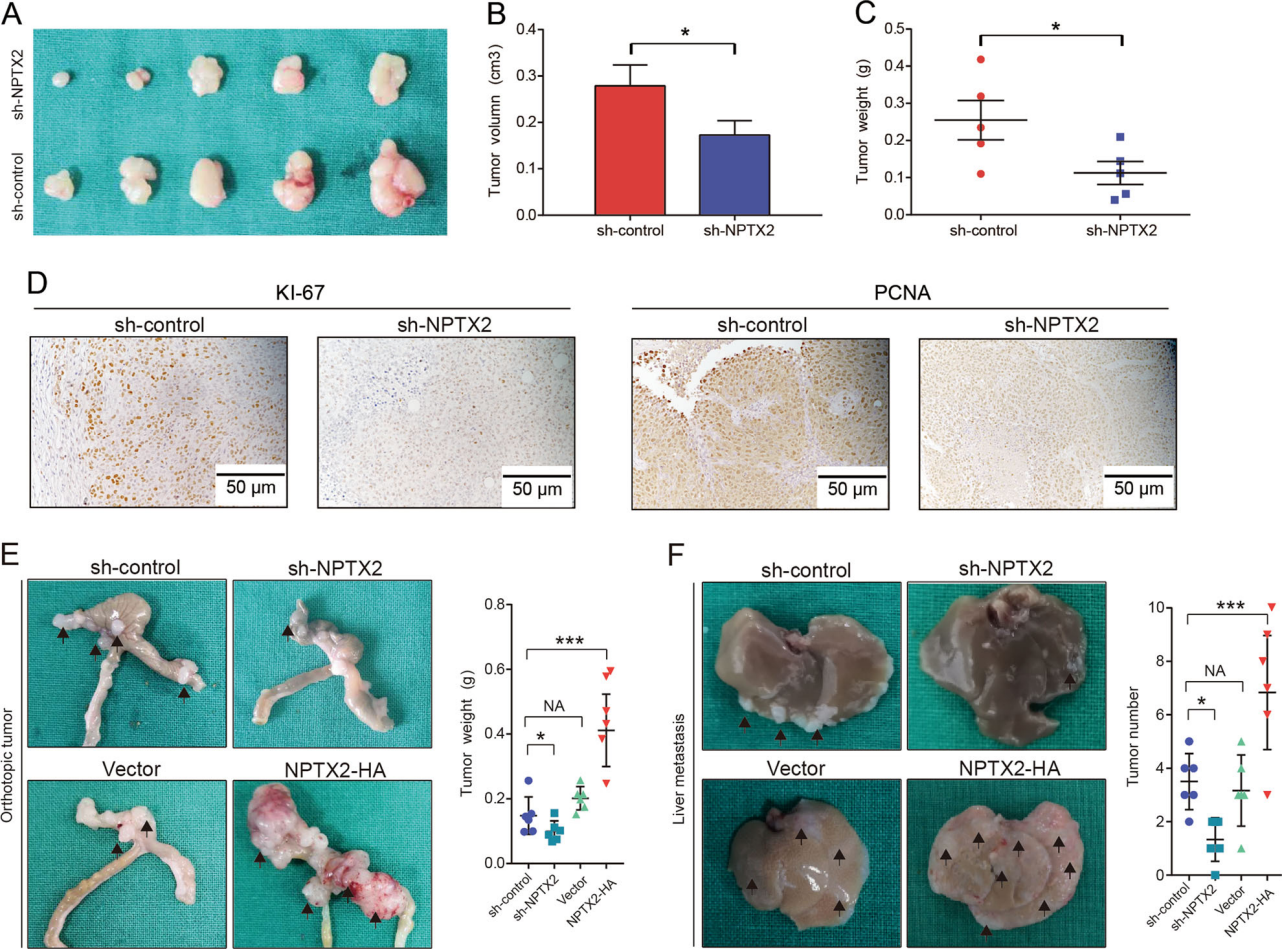
Knockdown of NPTX2 expression inhibits the proliferation and liver metastasis of CRC in vivo. **a** The proliferation of SW480 cells transfected with sh-NPTX2 or sh-control by subcutaneous tumor assay. **b** Comparison of subcutaneous tumor volume between sh-control group and sh-NPTX2 group (Student’s *t* test). **c** Comparison of subcutaneous tumor weight between sh-control group and sh-NPTX2 group (Student’s *t* test). **d** Expression of Ki-67 and PCNA in the subcutaneous tumor tissues (sh-control group [*n* = 5] and sh-NPTX2 group [*n* = 5]). **e** The proliferation of SW480 cells transfected with sh-NPTX2, sh-control, vector, and NPTX2-HA in an orthotopic mouse model of CRC (Student’s *t* test). **f** The proliferation of SW480 cells transfected with sh-NPTX2, sh-control, vector, and NPTX2-HA in a liver metastasis model of CRC (Student’s *t* test). All experiments had three replicates. Measurement data were presented as the mean ± SD. *P < 0.05

To explore the role of NPTX2 in CRC development in vivo, we applied three sets of experiments. First, sh-NPTX2 or sh-control CRC cells were subcutaneously transplanted into nude mice. As a result, the tumor volume and weight were significantly lower in the mice from sh-NPTX2 group than in those from sh-control group (Fig. 3a–c). We performed immunohistochemical staining for two markers of cell proliferation, namely, Ki-67 and PCNA, and found that the staining intensities for Ki-67 and PCNA were weaker in the tumor tissues obtained from NPTX2 knockdown cells than in those obtained from shRNA control cells (Fig. 3d). In the second set of experiment, we transplanted sh-NPTX2 or NPTX2-HA CRC cells into the cecum of nude mice to establish an orthotopic tumor model and found that the tumor weight significantly decreased after NPTX2 knockdown but obviously increased after NPTX2 overexpression (Fig. 3e). In addition, the overexpression of NPTX2 promoted ki-67 and PCNA expression, while NPTX2 knockdown inhibited ki-67 and PCNA expression in CRC (Fig. S2H, S2I). In the third experiment, we explored the role of NPTX2 in CRC metastasis; sh-NPTX2 or NPTX2-HA CRC cells were injected into the spleen of nude mice to simulate CRC metastasis to the liver through splenic vein-portal vein system. The results showed that the knockdown of NPTX2 expression inhibited CRC metastasis to the liver, while the overexpression of NPTX2 promoted CRC metastasis to the liver (Fig. 3f). Taken together, these data indicate that NPTX2 promotes CRC progression and metastasis to the liver.

### NPTX2 activates Wnt/β-catenin signaling and promotes nuclear translocation of β-catenin

**Fig. 4:**
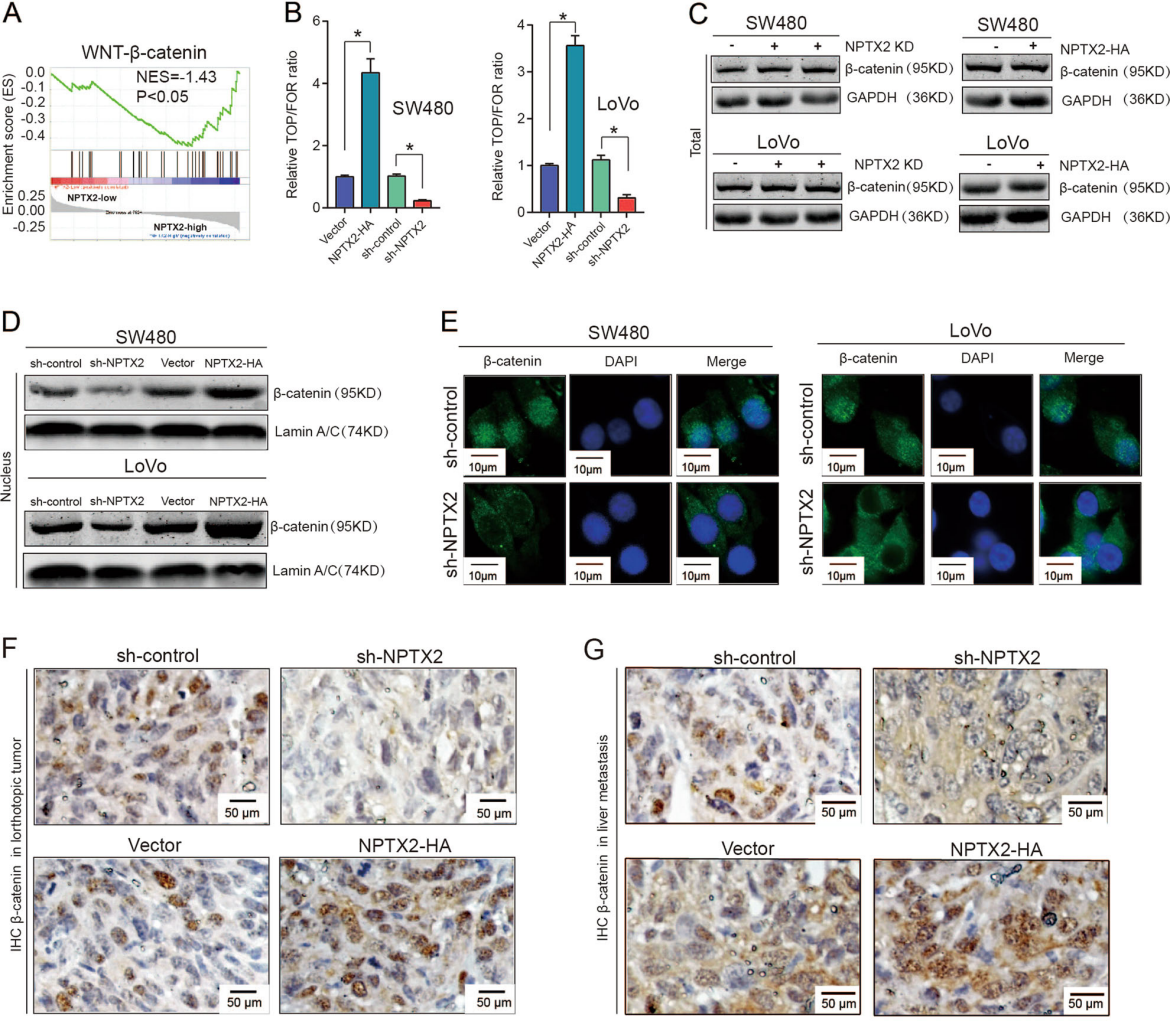
NPTX2 activates Wnt/β-catenin signaling and promotes the nuclear translocation of β-catenin. **a** GSEA analysis of NPTX2 expression in CRC, as evaluated from TCGA dataset. A total of 286 CRC samples were divided into two groups, including high NPTX2 expression group (143 samples) and low NPTX2 expression group (143 samples). **b** The fluorescence intensity of Wnt-β-catenin signaling after NPTX2 knockdown or overexpression was evaluated with a luciferase reporter assay. **c** Total β-catenin expression in SW480 and LoVo cells transfected with sh-NPTX2, sh-control, vector, and NPTX2-HA (Student’s *t* test). **d** Nuclear accumulation of β-catenin after NPTX2 knockdown and overexpression in SW480 and LoVo cells; **e** β-catenin localization in SW480 and LoVo cells transfected with sh-NPTX2 or sh-control. **f** β-catenin expression and localization in an orthotopic model of CRC injected with SW480 cells (sh-NPTX2 [*n* = 6], sh-control [*n* = 6], vector [*n* = 6], and NPTX2-HA [n = 6]). **g** β-catenin expression and localization in a liver metastasis model of CRC injected with SW480 cells (sh-NPTX2 [*n* = 6], sh-control [*n* = 6], vector [*n* = 6], and NPTX2-HA [*n* = 6]). *P < 0.05. All experiments were performed in triplicates. Measurement data were presented as the mean ± SD. *P < 0.05

To explore the mechanism underlying the NPTX2-mediated proliferation of CRC, we examined the expression of neuronal pentraxin receptor (NPTXR), the known receptor for NPTX2, in CRC tissues and cell lines. NPTXR was rarely expressed in CRC tissues and cell lines (Fig. S3A–S3C), indicating that some other unknown receptors or interaction partners of NPTX2 may play a role in this process. To reveal the mechanism underlying NPTX2 effects in CRC development, we conducted a Gene Set Enrichment Analysis (GSEA) analysis based on NPTX2 expression in CRC as per TCGA dataset, including the mRNA sequence data from patients with CRC. The samples were divided into two groups based on the expression level of NPTX2, including high expression of NPTX2 group (NPTX2 high group) and low expression of NPTX2 group (NPTX2 high group). The result showed that the gene sets from NPTX2 high expression group were enriched in Wnt/β-catenin signaling (Fig. 4a). To confirm the correlation between NPTX2 and Wnt/β-catenin pathways, we conducted a TOP/FOP luciferase activity assay and found that the knockdown of NPTX2 expression obviously inhibited the Wnt/β-catenin pathway, while NPTX2 overexpression activated the Wnt/β-catenin pathway in CRC (Fig. 4b and Fig. S4A). However, total β-catenin protein expression showed no significant change after NPTX2 knockdown or overexpression in SW480 and LoVo cell lines (Fig. 4c and Fig. S4B–S4E). As Wnt/β-catenin activation was associated with β-catenin translocation from the cytoplasm to the nucleus, we investigated whether NPTX2 could affect β-catenin translocation. The results showed that the knockdown of NPTX2 expression significantly reduced the nuclear localization of β-catenin, while the overexpression of NPTX2 promoted nuclear β-catenin localization in both SW480 and LoVo cell lines (Fig. 4d, e and Fig. S4F). We confirmed these results in the tumor tissues from orthotopic tumor models and liver metastasis models by immunohistochemical staining for β-catenin (Fig. 4f, g). Taken together, these data indicate that NPTX2 activates Wnt/β-catenin signaling and promotes the nuclear translocation of β-catenin.

### NPTX2 physically interacts with FZD6

**Fig. 5:**
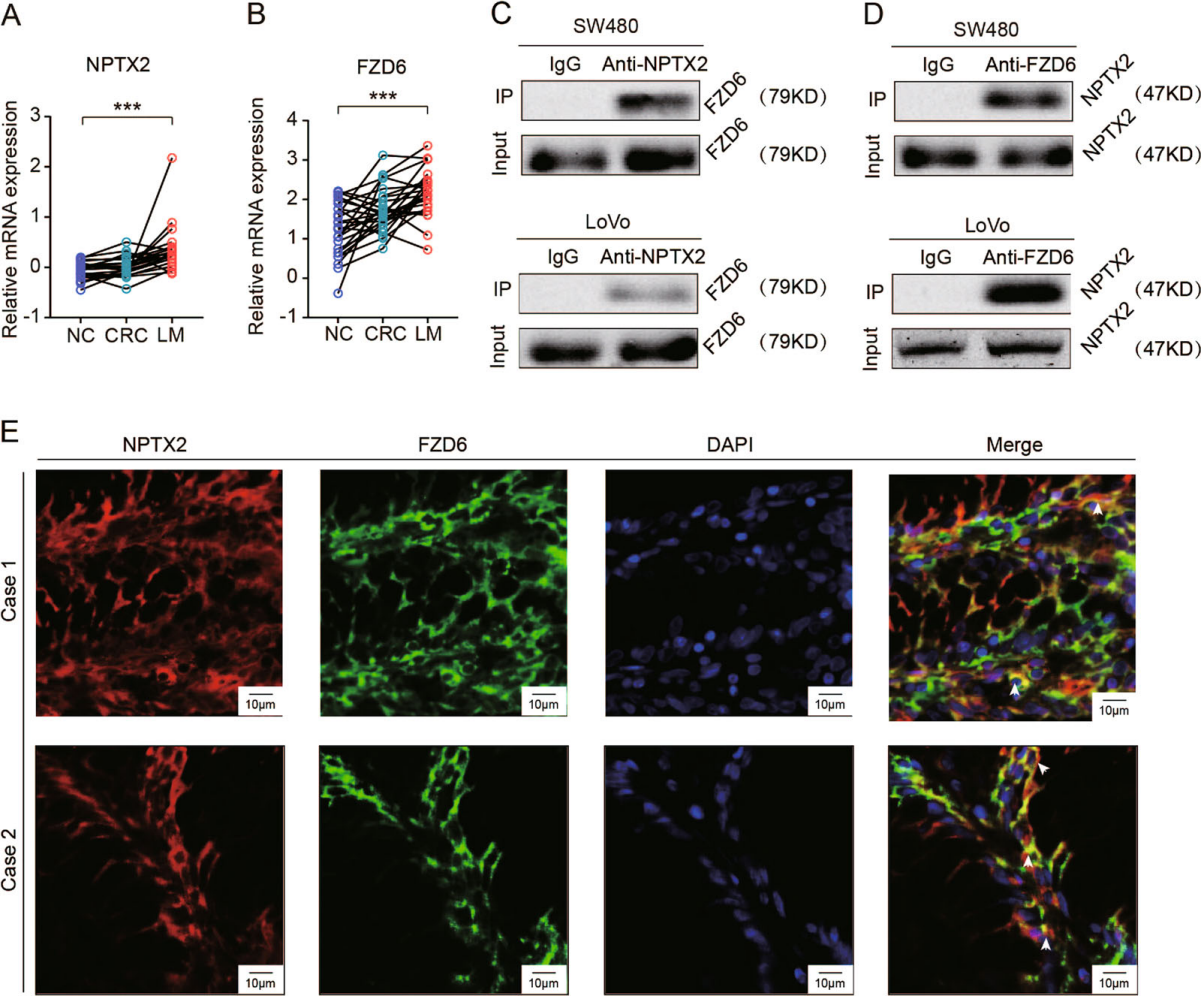
NPTX2 directly interacts with FZD6 in CRC. **a** The mRNA expression of FZD6 in normal colon tissue, CRC, liver metastasis (LM) analyzed by GSE6988 dataset (Student’s *t* test). **b** The mRNA expression of NPTX2 in normal colon tissue, CRC, liver metastasis (LM) analyzed by GSE6988 dataset (Student’s *t* test). **c**, **d** Co-IP of NPTX2 with FZD6, a receptor of the Wnt/β-catenin pathway, in SW480 and LoVo cells; **e** Co-localization of NPTX2 with FZD6 in CRC tissues, as analyzed with immunofluorescence assay. All experiments had three replicates. Measurement data were presented as the mean ± SD. ***P < 0.0001

As mentioned above, NPTXR expression was almost absent in CRC, indicating that NPTX2 activated the Wnt/β-catenin pathway in CRC via interaction with other unknown molecules. The FZD family of proteins plays a critical role in the Wnt/β-catenin pathway and interacts with extracellular ligands, including Wnt3a and Wnt5a, to regulate the Wnt/β-catenin pathway. Therefore, we focused on FZDs and investigated whether NPTX2 interacts with FZDs in CRC. By analysis of the expression pattern of FZDs and NPTX2 in GEO dataset (GSE6988), including normal colon tissue, CRC tissue, and liver metastasis, we found only FZD6 exhibited a similar expression pattern as NPTX2, which is gradually increase from normal colon tissue, CRC tissue to liver metastasis (Fig. 5a, b and Fig. S5A). To further validate whether FZD6 could directly interact with NPTX2, we performed a co-IP assay and found that NPTX2 physically interacted with FZD6 in both SW480 and LoVo cell lines (Fig. 5c, d and Fig. S5B). In addition, immunofluorescence assay showed that NPTX2 specifically co-localized with FZD6 in CRC tissues and cell lines (Fig. 5e and Fig. S5C), thereby confirming the interaction between NPTX2 and FZD6.

### FZD6 is indispensable for the NPTX2-mediated activation of Wnt/β-catenin signaling and CRC progression

**Fig. 6:**
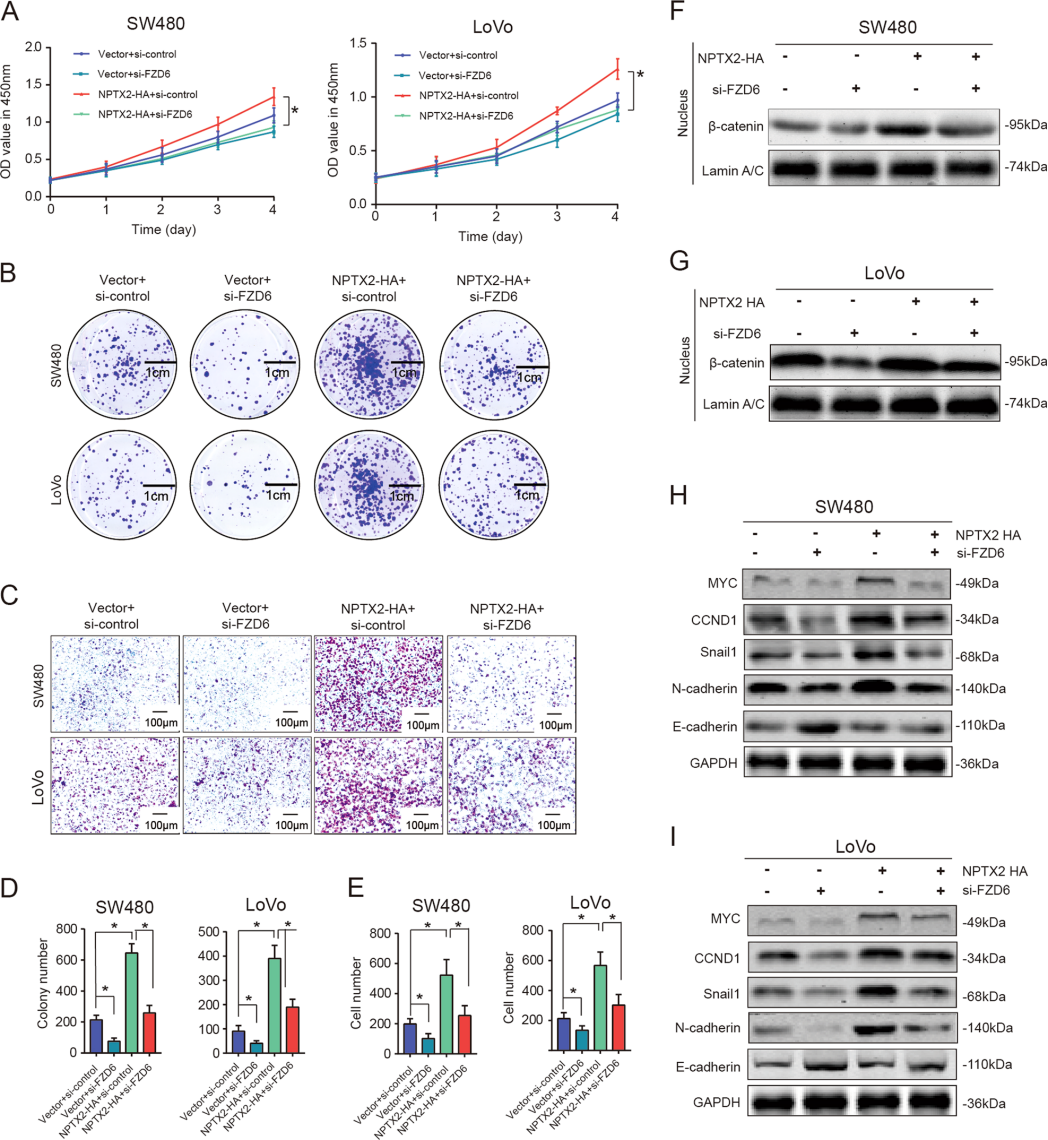
FZD6 is indispensable for the NPTX2-mediated activation of Wnt/β-catenin signaling in CRC. **a** Viability of NPTX2-HA SW480 and LoVo cells transfected with si-control and si-FZD6 (Student’s *t* test). **b** Colony-formation ability of NPTX2-HA SW480 and LoVo cells transfected with si-control and si-FZD6. **c** Cell migration ability of NPTX2-HA SW480 and LoVo cells transfected with si-control and si-FZD6. **d** Colony-formation ability of NPTX2-HA SW480 and LoVo cells transfected with si-control and si-FZD6 (Student’s *t* test). **e** Number of migratory cells among NPTX2-HA SW480 and LoVo cells transfected with si-control and si-FZD6 (Student’s *t* test). **f**, **g** β-catenin level in the nucleus of NPTX2-HA SW480 and LoVo cells transfected with si-control and si-FZD6. H and I. Protein expression of β-catenin target genes in NPTX2-HA SW480 and LoVo cells transfected with si-control and si-FZD6. In all the graphs relative to experiments with cell lines, three replicates were evaluated. Measurement data were presented as the mean ± SD. *P < 0.05

Based on the interaction between NPTX2 and FZD6, we investigated whether FZD6 serves as a key molecule in the NPTX2-mediated regulation of CRC development. The positive effects of NPTX2 on CRC proliferation and migration were almost completely abolished after the knockdown of FZD6 expression (Fig. 6a–e). Furthermore, the NPTX2-mediated nuclear localization of β-catenin also significantly reduced after the knockdown of FZD6 expression (Fig. 6f, g). The expression of some downstream target genes of Wnt/β-catenin, such as MYC, CCND1, snail-1, and N-cadherin, obviously increased after the overexpression of NPTX2, while these effects were significantly inhibited after FZD6 knockdown. E-cadherin protein expression obviously reduced after the overexpression of NPTX2, but this effect was completely abolished by FZD6 knockdown (Fig. S6A–S6E and Fig. 6h, i). Taken together, these data indicate that NPTX2 promoted CRC cell proliferation and metastasis through the activation of the Wnt/β-catenin pathway via direct interaction with FZD6.

## Discussion

Although there is no consensus on the relationship between nervous system diseases and cancer, the overall risk of cancer was shown to decrease in the parents and siblings of the patients with nervous system diseases^14^. Some researchers suggest that the patients with Alzheimer’s and Parkinson’s diseases have low risk of CRC^15,16^. Several molecules have been found to exhibit opposite expression patterns between cancer and nervous system diseases, including TP53, adenomatous polyposis coli (APC), neuregulin-I, and phosphoinositide 3-kinase (PI3K)^17^. These interesting discoveries indicate that a series of tumor-related genes and nerve-related molecules show inverse expression patterns.

Another study analyzed the differential expression of genes between Alzheimer’s and non-Alzheimer’s diseases and found that the expression of C-Met, NPTX2, neurogenic differentiation factor 6 (NEUROD6), hyperpolarization-activated cyclic nucleotide-gated potassium channel 1 (HCN1), etc., significantly decreased in Alzheimer’s disease^18^. Xiao et al.^19^ found that NPTX2 expression decreased in Alzheimer’s disease and closely correlated with cognitive functions. Li et al.^20^ reported the downregulation of NPTX2 expression in female mice with anxiety and depression. In the present study, NPTX2 was overexpressed in CRC tissues, and high NPTX2 expression positively associated with T stages, lymph node invasion, distant metastasis, pathological stage, and poor outcomes of patients with CRC. A study evaluating the relationship between NPTX2 expression and cancer prognosis found that NPTX2 was overexpressed in human neuroblastoma stage IV and was positively associated with poor prognosis^8^. Moreover, we found NPTX2 expression was higher in CRC liver metastasis than that in primary CRC tissue. Our data showed NPTX2 could promote CRC liver metastasis and enhance cell migration and proliferation abilities. These data proved that NPTX2-expressing cells had stronger migration abilities to the liver, resulting in higher expression of NPTX2 in CRC liver metastasis. Another possibility is that the metastasized CRC could secret more NPTX2 to support their colonization (growth, metastasis) by activating β-catenin signaling. It has been reported that β-catenin signaling is very important for the colonization of liver metastasis of CRC^21,22^.

NPTX2 as an extracellular ligand binds to its receptor, NPTXR. Extensive studies have revealed the importance of the NPTX2/NPTXR axis in nervous system diseases, involving recruitment of glutamate receptors and formation of synapses. The NPTX2/NPTXR system was shown to be upregulated in neuroblastoma and promote tumor development^10^. In the present study, we found that NPTX2 expression was significantly upregulated in CRC, while, to our surprise, NPTXR expression was almost completely absent in CRC. GSEA analysis revealed the close correlation between NPTX2 and Wnt/β-catenin signaling. The Wnt/β-catenin pathway is a vital regulatory pathway in CRC, and several studies have highlighted the close association between the Wnt pathway and neurite outgrowth^23^. Here, we demonstrate for the first time that NPTX2 promoted CRC progression and liver metastasis through the activation of Wnt/β-catenin signaling.

FZD family of proteins is the most important receptor family of the Wnt/β-catenin pathway, and abnormal expression of FZDs was closely related to carcinogenesis, especially in CRC^24,25^. Moreover, FZD3, FZD6, and FZD7 play vital roles in CRC tumorigenesis^26–28^. The Wnt protein family is a large family of ligands involved in the Wnt/β-catenin pathway, and Wnt3a and Wnt5a are the most important ligands in CRC. Wnt3a activates the Wnt-β-catenin pathway mainly through its interaction with FZD2/4/5^29^. Wnt5a may regulate the Wnt-β-catenin pathway by interacting with FZD2/5/6^30,31^. Aside from the Wnt family, others ligands were found to bind with FZDs to regulate the Wnt/β-catenin pathway^32–34^. We found that the mRNA expression of FZD6 was gradually upregulated from normal colon tissue, CRC tissue to liver metastasis, which was similar to the expression pattern of NPTX2. Subsequent, we found that NPTX2 interacted with FZD6 in CRC and demonstrated that FZD6 is indispensable for the NPTX2-mediated activation of the Wnt/β-catenin signaling and CRC progression.

There are a few limitations associated with this study. First, as the Wnt-/β-catenin pathway is a very complex system, we only revealed the interaction between NPTX2 and FZD6, but did not identify the effects of NPTX2 on other receptors or ligands, such as Wnt ligand family. Second, as an extracellular protein, NPTX2 may regulate the tumor microenvironment of CRC. For instance, the intestinal tract is rich in enteric nerve cells that may be affected by NPTX2 expression. Therefore, it would be interesting to investigate the correlation between NPTX2 expression and enteric nerve cells in CRC microenvironment.

Taken together, the present study revealed a novel molecule involved in CRC development and highlighted the inverse correlation between individual risk of cancer and nervous system diseases, owing to the differential expression of nerve-related molecules such as NPTX2.

## Supplementary information

revised supplementary material

Figure S1

Figure S2

Figure S3

Table S1

Table S2

Figure S4

Figure S5

Figure S6
