# Supplementary material for: NPTX2 promotes colorectal cancer growth and liver metastasis by the activation of the canonical Wnt/β-catenin pathway via FZD6
Source: Cell Death Dis. 2019 Mar 4;10(3):217. doi: 10.1038/s41419-019-1467-7 (PMC6399240; doi:10.1038/s41419-019-1467-7)
Supplement: Supplementary file 1 — revised supplementary material [file 41419_2019_1467_MOESM1_ESM.docx]

**Supplementary material**

**Supplementary figure and legends**

Figure S1


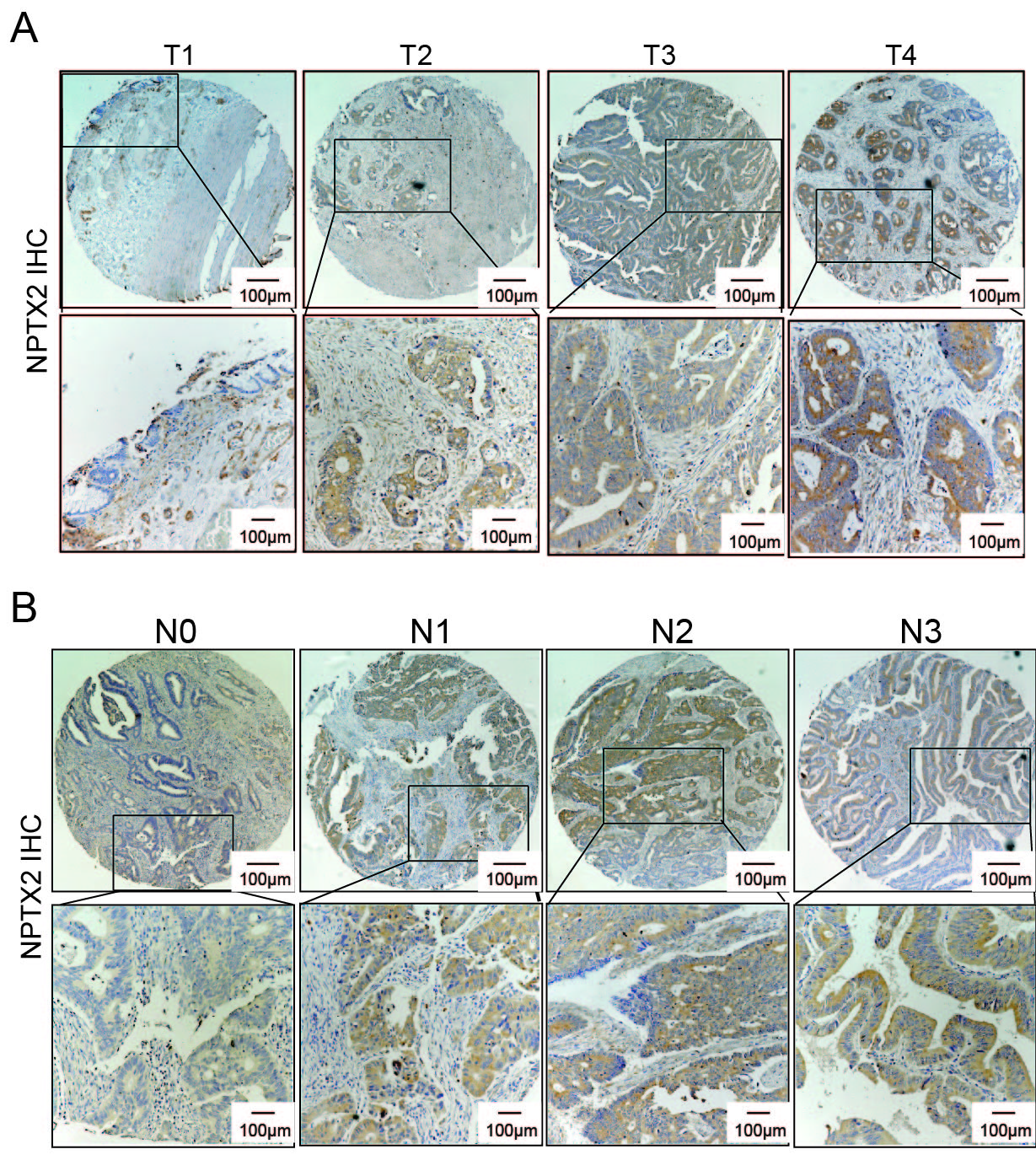


Figure S1. The protein expression of NPTX2 in different T stages and N stages of CRC patients in tissue microarray. A. The protein expression of NPTX2 in different T stages of CRC patients in tissue microarray (T1: n = 11; T2: n = 36: T3: n = 94; T4: n = 251.); B. NPTX2 protein expression in different lymph node invasion stages of CRC patients in tissue microarray (N0: n = 211; N1: n =90; N2: n = 71; N3: n = 20).

Figure S2


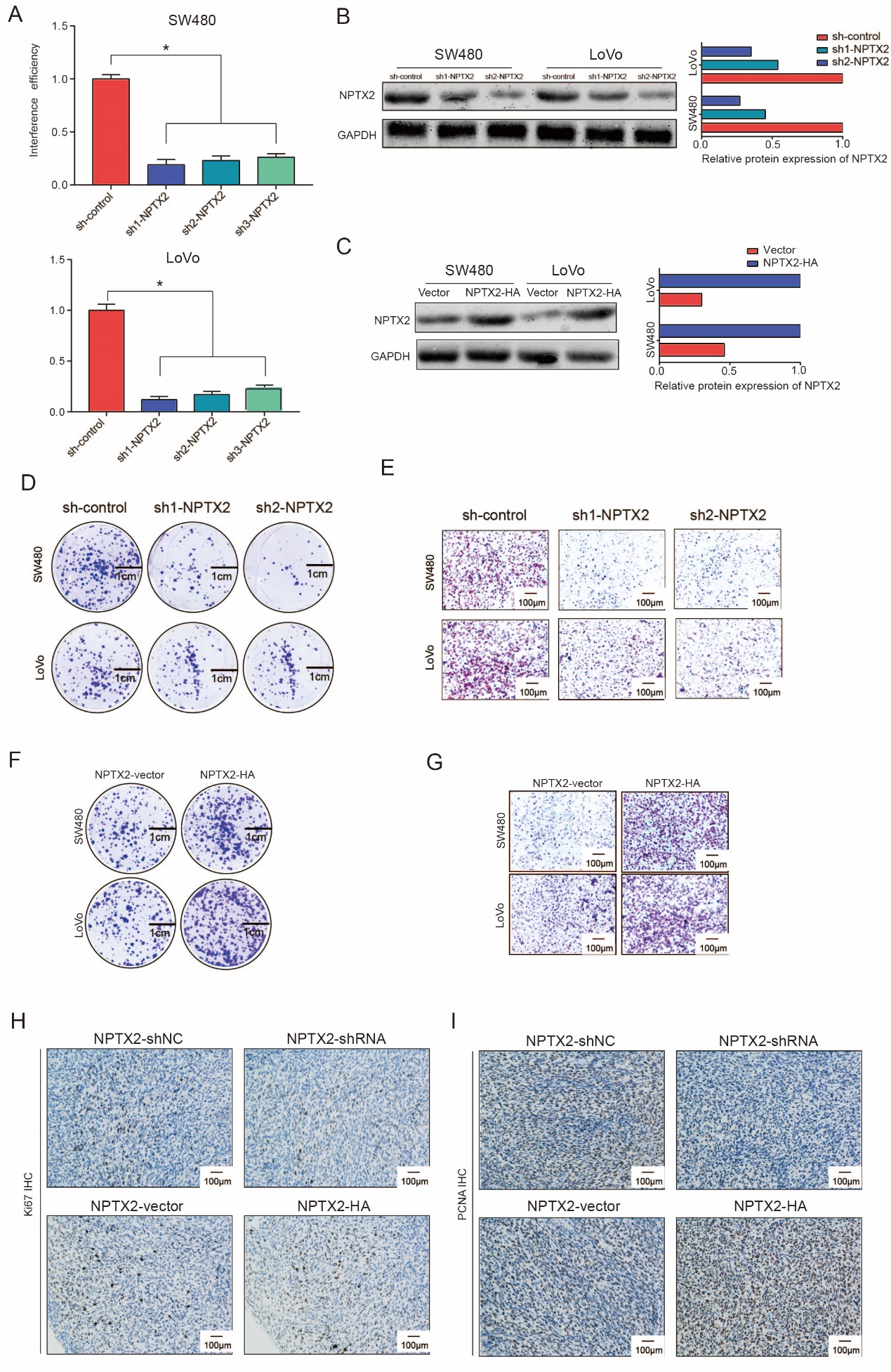


Figure S2. Overexpression of NPTX2 promotes cell viability, colony formation and cell migration in CRC cell lines. A and B. Interference efficiency of NPTX2 in SW480 and LoVo cell; C. NPTX2 overexpression in SW480 and LoVo cell; D. The colony formation of SW480 and LoVo cells transfected with sh-NPTX2 or sh-control; E. The cell migration ability of SW480 and LoVo cell transfected with sh-NPTX2 or sh-control; F. The colony formation of SW480 and LoVo cells transfected with vector or NPTX2-HA; G. The cell migration ability of SW480 and LoVo cell transfected with vector or NPTX2-HA. H and I. Ki-67 and PCNA expression after NPTX2 knockdown or overexpression in orthotopic mouse model of CRC; In all the graphs relative to experiments with cell lines, three replicates have been evaluated to experimental result. Measurement data were presented as the mean ± SD. Student’s *t*-test was used to statistical analysis. *P < 0.05.

Figure S3


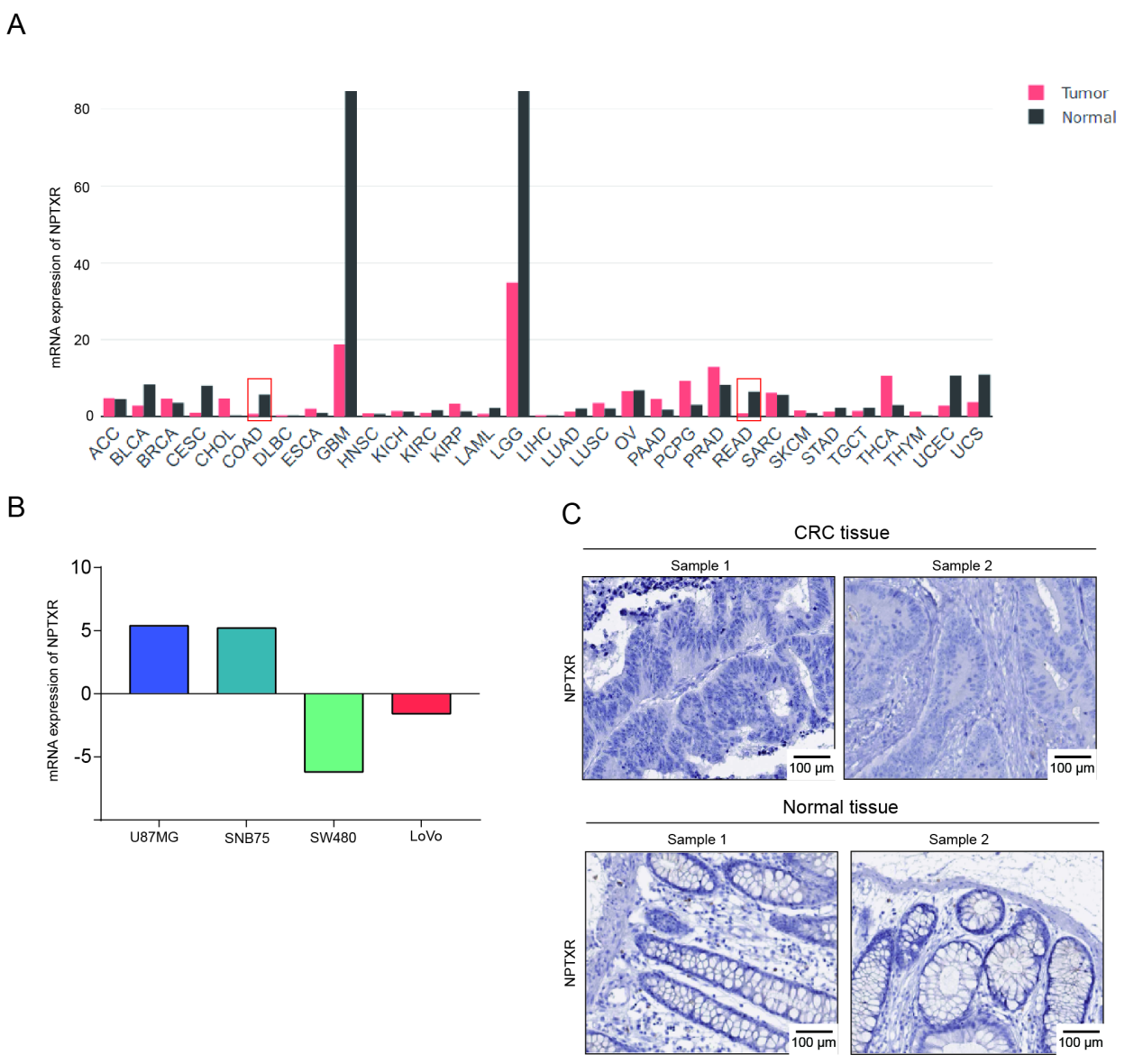


Figure S3. NPTXR expression in CRC. A. The mRNA expression of NPTXR in tumors from GEPIA (<http://gepia.cancer-pku.cn/index.html>); B. The mRNA expression of NPTXR in cancer cell lines from CCLE (<https://portals.broadinstitute.org/ccle>), CRC cell line: SW480 and LoVo，Glioblastoma cell line:U87MG and SNB75; C. The protein expression of NPTXR in CRC tissue from proteinatlas (<https://www.proteinatlas.org/>).

Figure S4


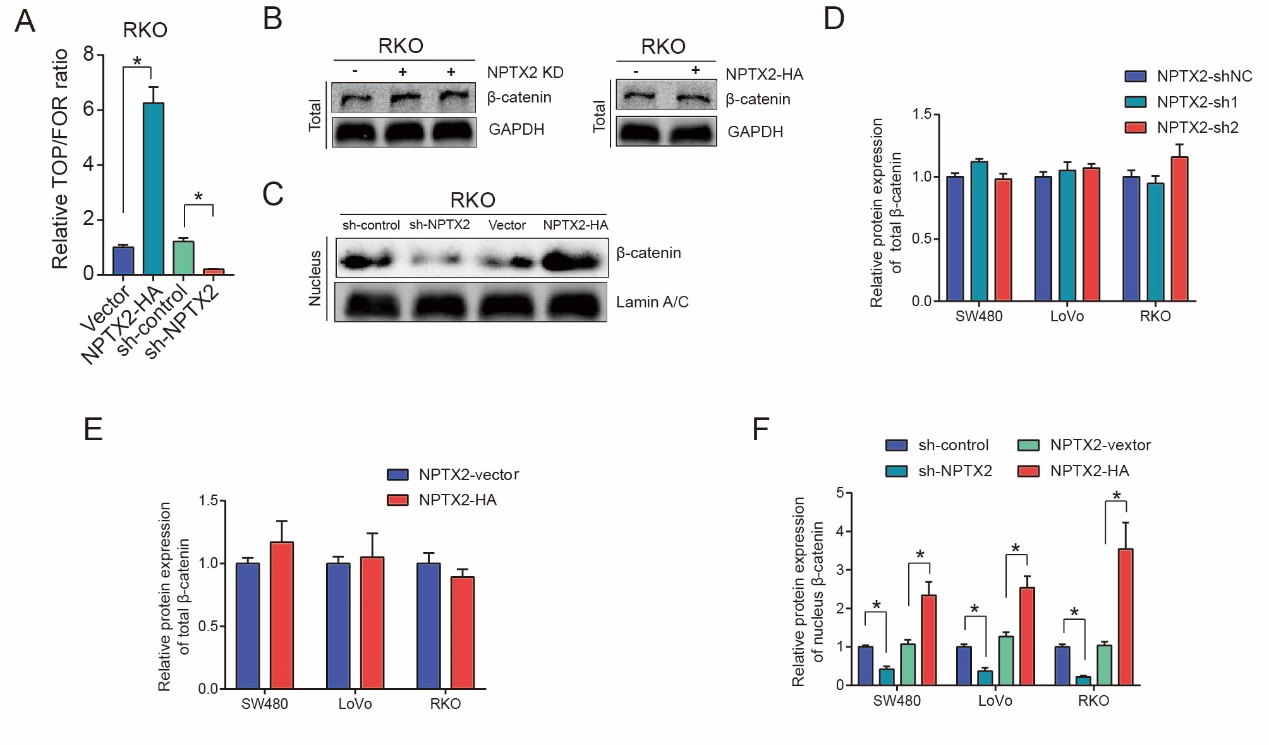


Figure S4. NPTX2 promotes β-catenin into nucleus in CRC. A. The fluorescence intensity of Wnt-β-catenin signal after NPTX2 knockdown or overexpression by Luciferase reporter assay; B. Western blot for total β-catenin expression of RKO cells transfected with sh-NPTX2, sh-control, Vector and NPTX2-HA, respectively; C. Western blot for nucleus β-catenin accumulation after NPTX2 knockdown and overexpression in RKO cells; D. Total β-catenin expression after NPTX2 knockdown in SW480, LoVo and RKO cells; E. Total β-catenin expression after NPTX2 over-expression in SW480, LoVo and RKO cells; F. Nucleus β-catenin accumulation after NPTX2 knockdown or overexpression in SW480, LoVo and RKO cells; In all the graphs relative to experiments with cell lines, all experiments had three replicates. Measurement data were presented as the mean ± SD. Student’s *t*-test was used to statistical analysis. *P < 0.05.

Figure S5


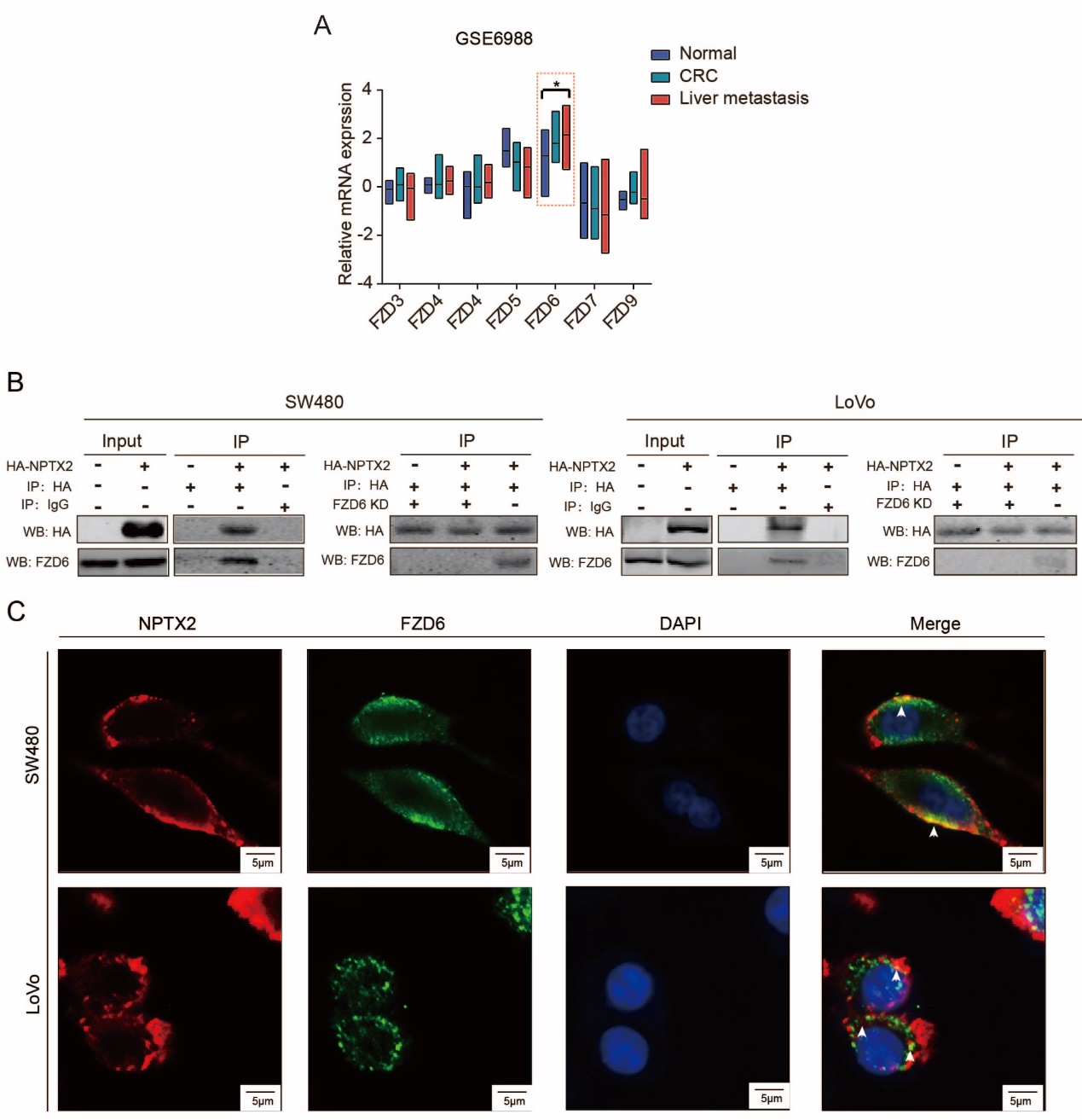


Figure S5. Interaction of NPTX2 with FZD6 in CRC. A. The mRNA expression of FZD6 in normal colon tissue, CRC, liver metastasis (LM) analyzed by GSE6988 dataset (Student’s *t*-test). B. Co-IP of NPTX2 with FZD6, a receptor of Wnt/β-catenin pathway, in SW480 and LoVo cells; C. Co-localization of NPTX2 with FZD6 in SW480 and LoVo cells by immunofluorescence assay. All experiments had three replicates. Measurement data were presented as the mean ± SD. * P < 0.05.

Figure S6


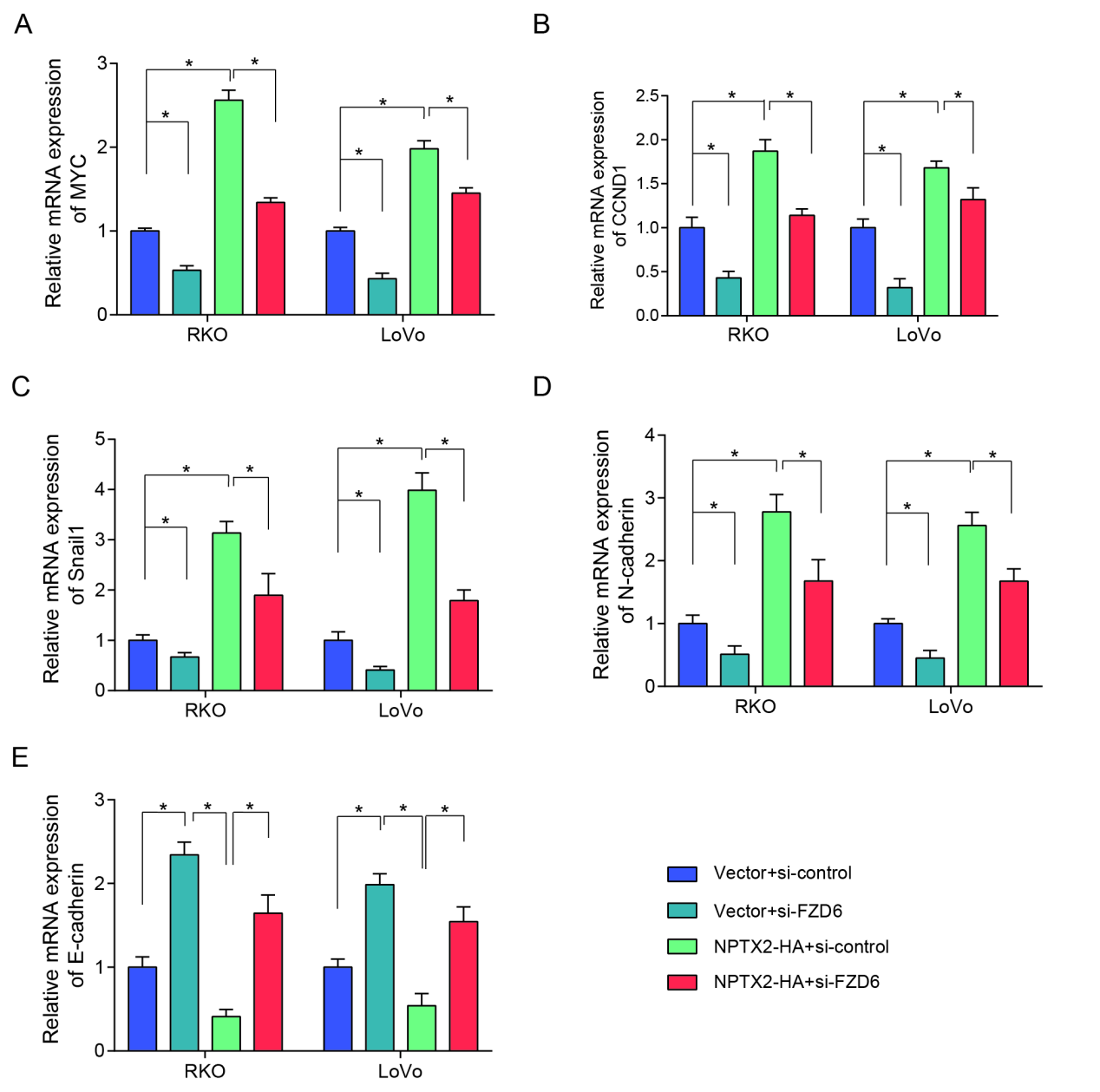


Figure S6. FZD6 is indispensable for NPTX2 regulating downstream genes of Wnt/β-catenin pathway. A. The mRNA expression of MYC in NPTX2-HA SW480 and LoVo cell with si-control and si-FZD6; B. The mRNA expression of CCND1 in NPTX2-HA SW480 and LoVo cell with si-control and si-FZD6; C. The mRNA expression of Snail1 in NPTX2-HA SW480 and LoVo cell with si-control and si-FZD6; D. The mRNA expression of N-cadherin in NPTX2-HA SW480 and LoVo cell with si-control and si-FZD6; E. mRNA expression of E-cadherin in NPTX2-HA SW480 and LoVo cell with si-control and si-FZD6. All experiments had three replicates. Measurement data were presented as the mean ± SD. Student’s *t*-test was used to statistical analysis. *P < 0.05.
