# Supplementary figures and images for: NPTX2 promotes colorectal cancer growth and liver metastasis by the activation of the canonical Wnt/β-catenin pathway via FZD6
Source: Cell Death Dis. 2019 Mar 4;10(3):217. doi: 10.1038/s41419-019-1467-7 (PMC6399240; doi:10.1038/s41419-019-1467-7)

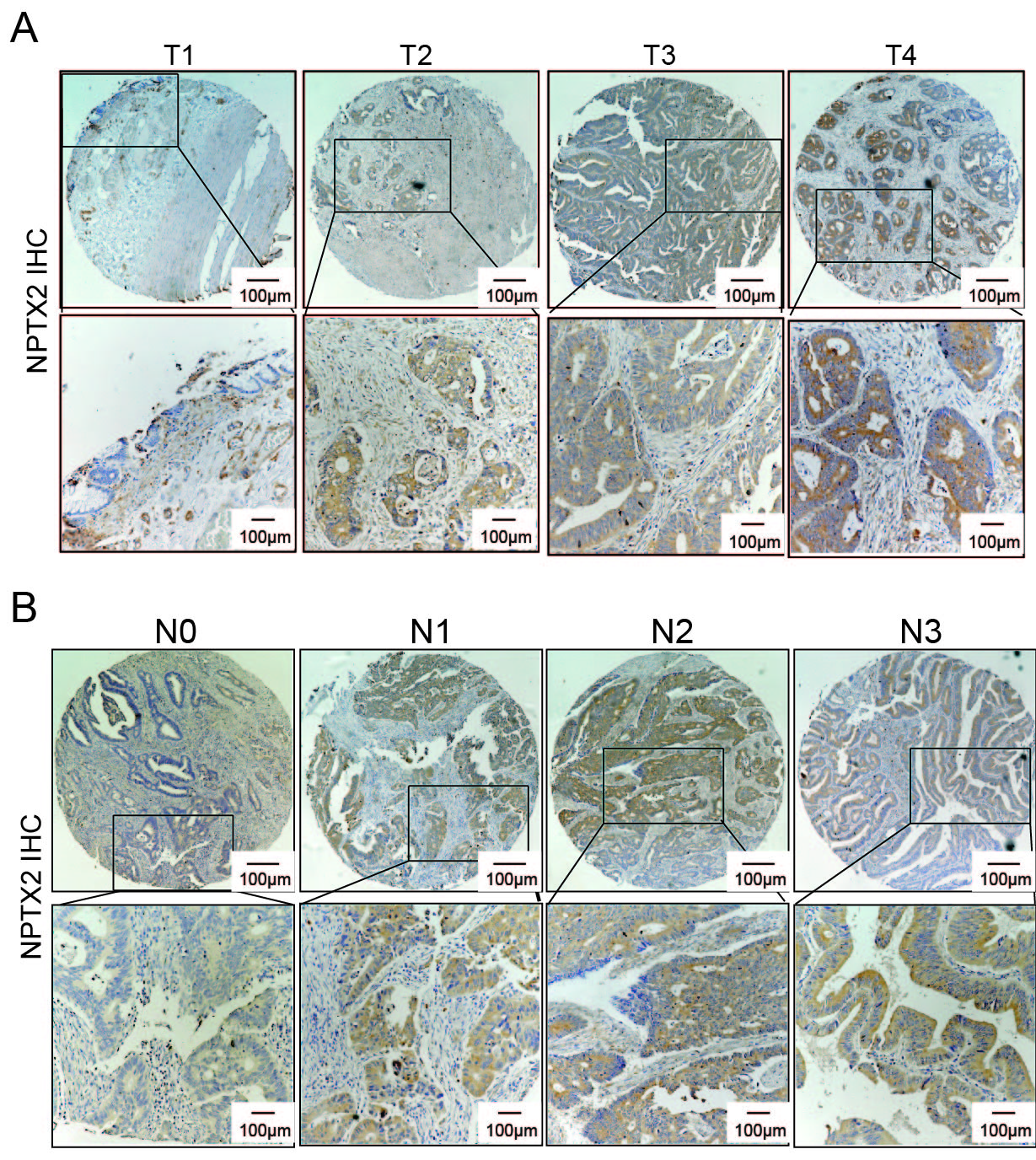

Supplement: Supplementary file 2 — Figure S1 [file 41419_2019_1467_MOESM2_ESM.jpg]

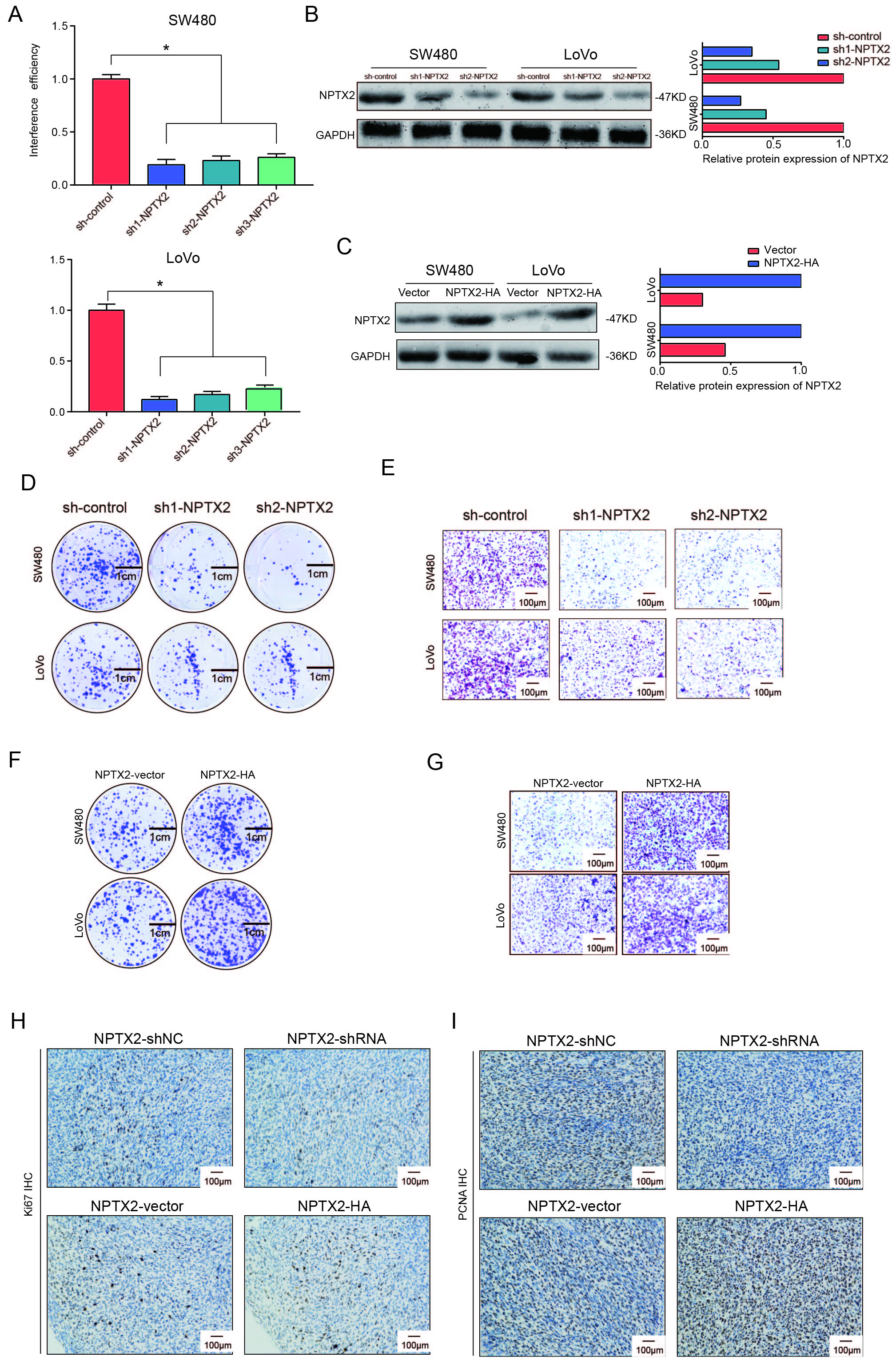

Supplement: Supplementary file 3 — Figure S2 [file 41419_2019_1467_MOESM3_ESM.jpg]

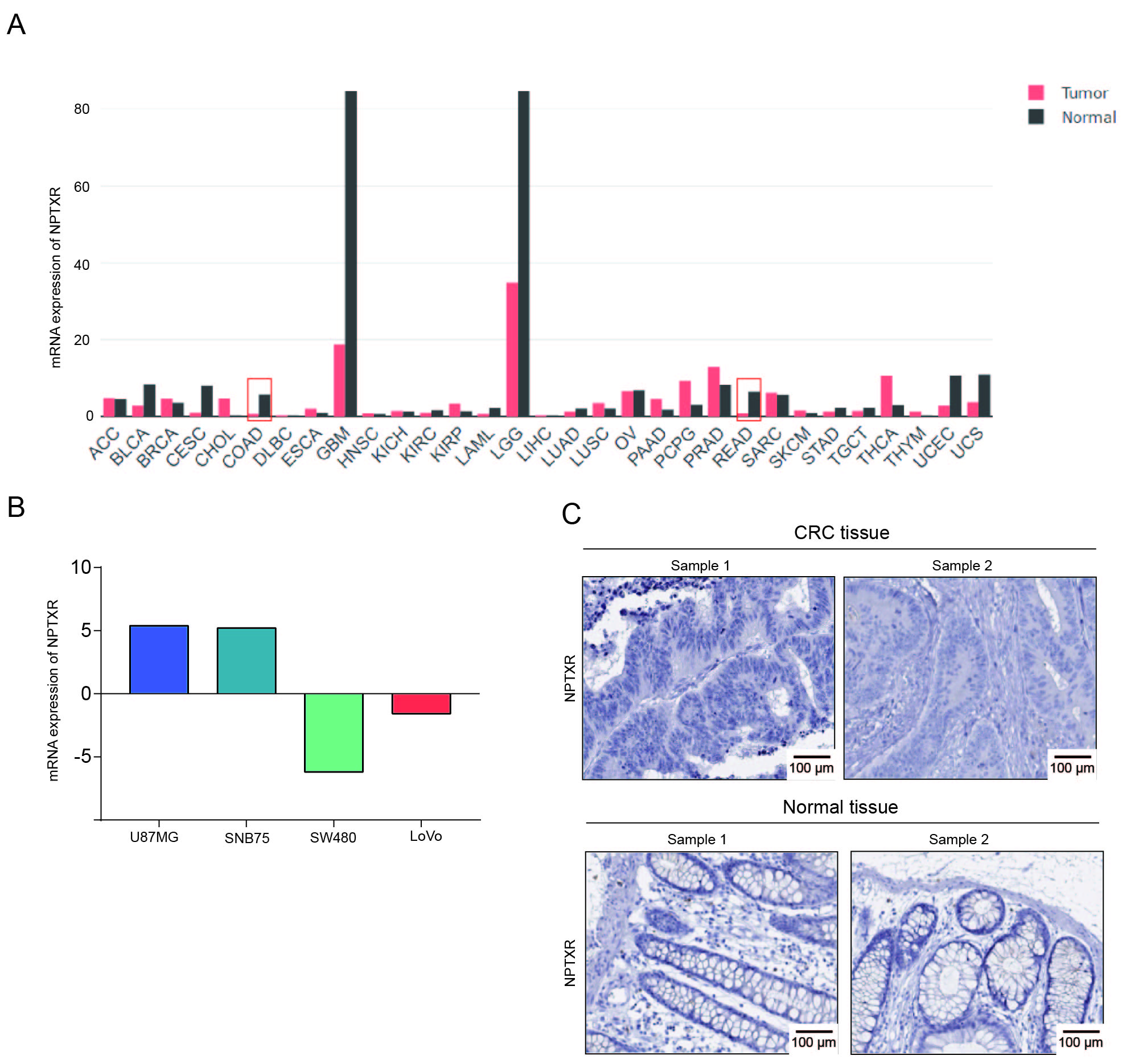

Supplement: Supplementary file 4 — Figure S3 [file 41419_2019_1467_MOESM4_ESM.jpg]

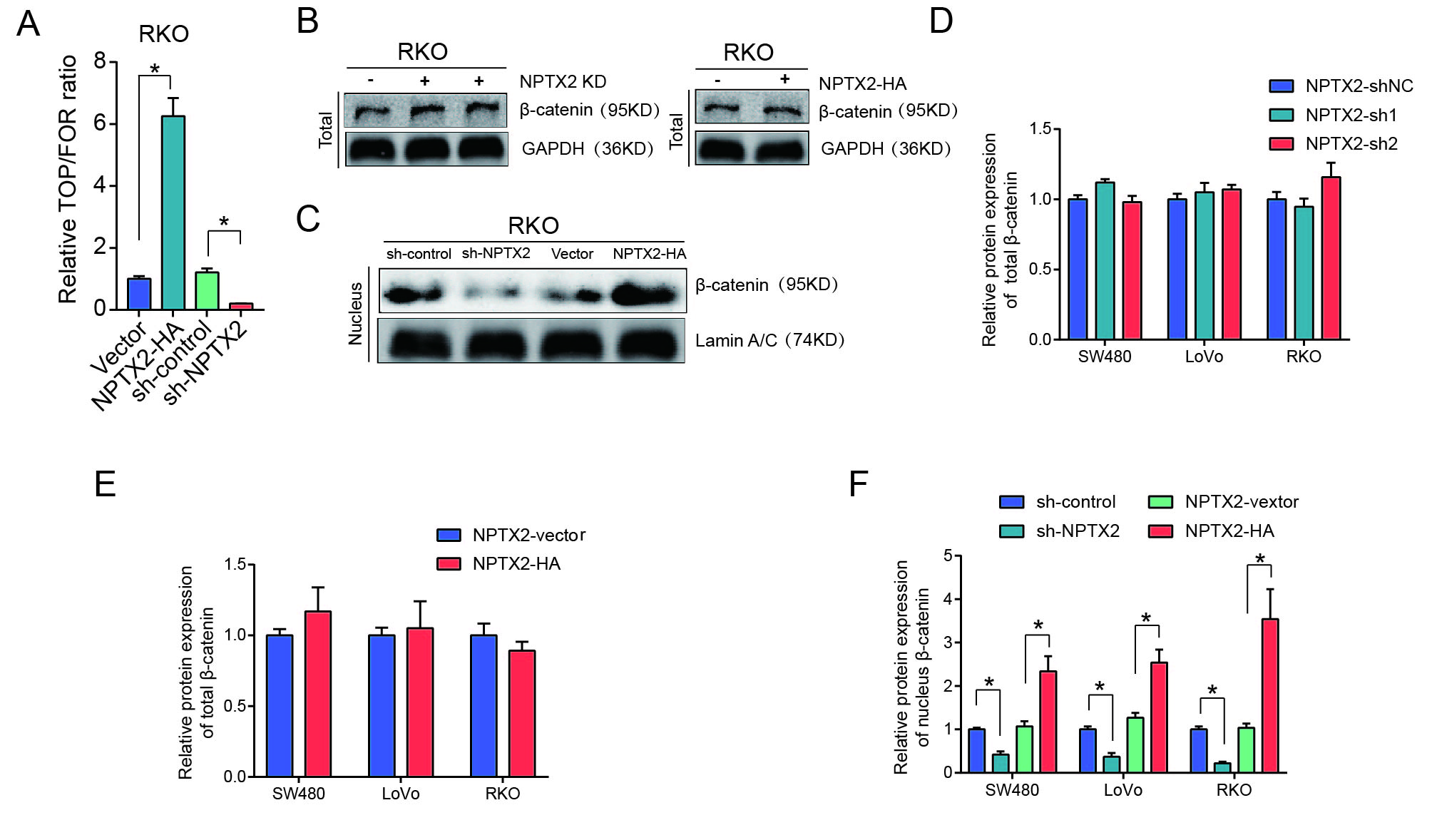

Supplement: Supplementary file 7 — Figure S4 [file 41419_2019_1467_MOESM7_ESM.jpg]

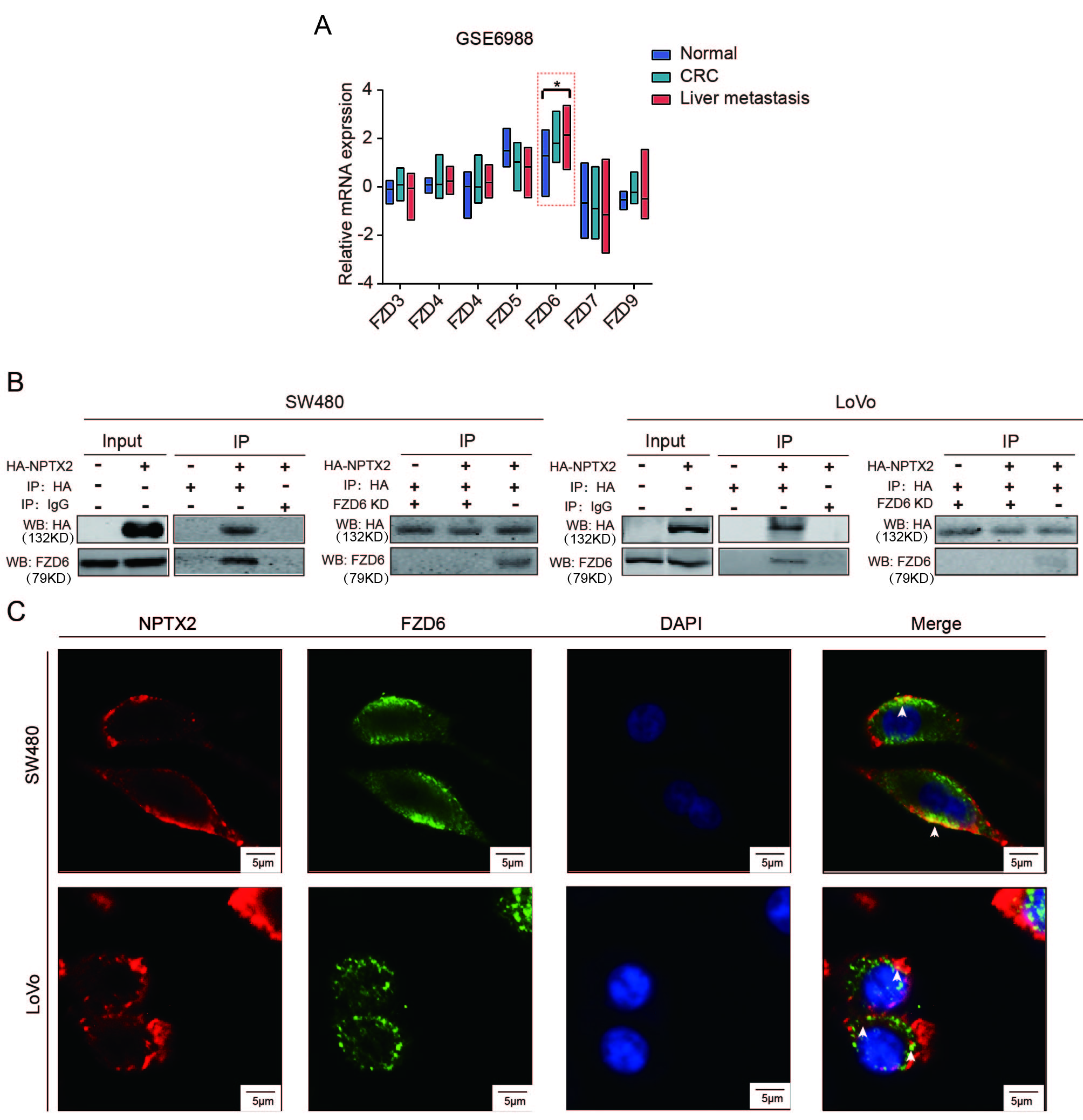

Supplement: Supplementary file 8 — Figure S5 [file 41419_2019_1467_MOESM8_ESM.jpg]

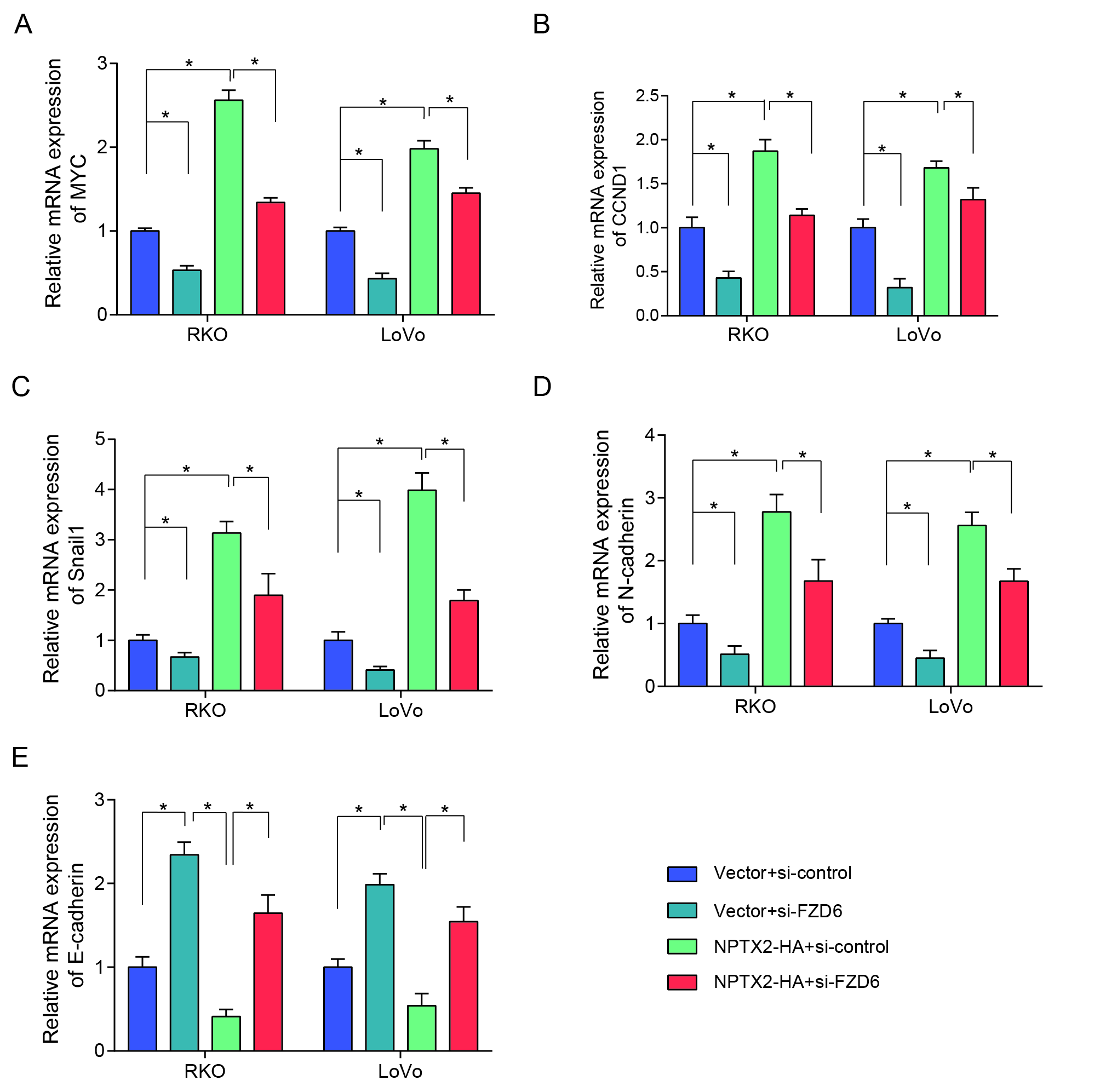

Supplement: Supplementary file 9 — Figure S6 [file 41419_2019_1467_MOESM9_ESM.tif]
