# Supplementary material for: NPTX2 promotes colorectal cancer growth and liver metastasis by the activation of the canonical Wnt/β-catenin pathway via FZD6
Source: Cell Death Dis. 2019 Mar 4;10(3):217. doi: 10.1038/s41419-019-1467-7 (PMC6399240; doi:10.1038/s41419-019-1467-7)
Supplement: Supplementary file 5 — Table S1 [file 41419_2019_1467_MOESM5_ESM.docx]

Table S1 siRNA sequence

|  | | **sequence** |
| --- | --- | --- |
|  |  |  |
| **NPTX2 siNC** | **sense** | 5'-UUCUUCGAACGUGUCACGUTT-3' |
|  | **antisense** | 5'-ACGUGACACGUUCGGAGAATT-3' |
| **NPTX2 si1** | **sense** | 5'-GCGAGGCAAUAGCGCCUUUTT-3' |
|  | **antisense** | 5'-AAAGGCGCUAUUGCCUCGCTT-3' |
| **NPTX2 si2** | **sense** | 5'-GCUCAUCAACGACAAGGUUTT-3' |
|  | **antisense** | 5'-AACCUUGUCGUUGAUGAGCTT-3' |
| **NPTX2 si3** | **sense** | 5'-CCUUCGCGCACAAGAAAUUTT-3' |
|  | **antisense** | 5'-AAUUUCUUGUGCGCGAAGGTT-3' |
| **FZD6 siNC** | **sense** | 5'-UUCUCCGAACGUGUCACGUTT-3' |
|  | **antisense** | 5'-ACGUGACACGUUCGGAGAATT-3' |
| **FZD6 si1** | **sense** | 5'-GCAAUAGUACAGCCUGCAATT-3' |
|  | **antisense** | 5'-UUGCAGGCUGUACUAUUGCTT-3' |
| **FZD6 si2** | **sense** | 5'-GCUGGCAUUAUUUCCUUAATT-3' |
|  | **antisense** | 5'-UUAAGGAAAUAAUGCCAGCTT-3' |
| **FZD6 si3** | **sense** | 5'-CCAUUGUCGUCAGUACCAUTT-3' |
|  | **antisense** | 5'-AUGGUACUGACGACAAUGGTT-3' |
