# Supplementary material for: NPTX2 promotes colorectal cancer growth and liver metastasis by the activation of the canonical Wnt/β-catenin pathway via FZD6
Source: Cell Death Dis. 2019 Mar 4;10(3):217. doi: 10.1038/s41419-019-1467-7 (PMC6399240; doi:10.1038/s41419-019-1467-7)
Supplement: Supplementary file 6 — Table S2 [file 41419_2019_1467_MOESM6_ESM.docx]

Table S2 the relationship between NPTX2 mRNA expression and clinical features in CRC from TCGA dataset.

|  |  | **NPTX2** | | | | ***P*** value | |
| --- | --- | --- | --- | --- | --- | --- | --- |
|  |  | **Low**（n=50） | | **High(n=50)** | |  |  |
| **T stage** | |  |  |  |  | **<0.01** | |
| T1 | | 4 | | 0 | |  |  |
| T2 | | 11 | | 4 | |  |  |
| T3 | | 27 | | 39 | |  |  |
| T4 | | 8 | | 7 | |  |  |
| **Lymph node invasion** | |  |  |  |  |  |  |
| yes | | 33 | | 21 | | **0.016** | |
| no | | 17 | | 29 | |  |  |
| **Distant metastasis** | |  |  |  |  |  |  |
| yes | | 2 | | 10 | | **0.042** | |
| no | | 48 | | 40 | |  |  |
| **Pathological staging** | |  |  |  |  |  |  |
| Ⅰ | | 15 | | 2 | | **<0.001** | |
| Ⅱ | | 18 | | 15 | |  |  |
| Ⅲ | | 15 | | 23 | |  |  |
| Ⅳ | | 2 | | 10 | |  |  |

NOTE: Chi-square analysis was applied to statistical analysis.
